# The Groucho Co-repressor Is Primarily Recruited to Local Target Sites in Active Chromatin to Attenuate Transcription

**DOI:** 10.1371/journal.pgen.1004595

**Published:** 2014-08-28

**Authors:** Aamna Kaul, Eugene Schuster, Barbara H. Jennings

**Affiliations:** 1 UCL Cancer Institute, University College London, London, United Kingdom; 2 Department of Genetics, Evolution and Environment, University College London, London, United Kingdom; University of Cambridge, United Kingdom

## Abstract

Gene expression is regulated by the complex interaction between transcriptional activators and repressors, which function in part by recruiting histone-modifying enzymes to control accessibility of DNA to RNA polymerase. The evolutionarily conserved family of Groucho/Transducin-Like Enhancer of split (Gro/TLE) proteins act as co-repressors for numerous transcription factors. Gro/TLE proteins act in several key pathways during development (including Notch and Wnt signaling), and are implicated in the pathogenesis of several human cancers. Gro/TLE proteins form oligomers and it has been proposed that their ability to exert long-range repression on target genes involves oligomerization over broad regions of chromatin. However, analysis of an endogenous *gro* mutation in *Drosophila* revealed that oligomerization of Gro is not always obligatory for repression *in vivo*. We have used chromatin immunoprecipitation followed by DNA sequencing (ChIP-seq) to profile Gro recruitment in two *Drosophila* cell lines. We find that Gro predominantly binds at discrete peaks (<1 kilobase). We also demonstrate that blocking Gro oligomerization does not reduce peak width as would be expected if Gro oligomerization induced spreading along the chromatin from the site of recruitment. Gro recruitment is enriched in “active” chromatin containing developmentally regulated genes. However, Gro binding is associated with local regions containing hypoacetylated histones H3 and H4, which is indicative of chromatin that is not fully open for efficient transcription. We also find that peaks of Gro binding frequently overlap the transcription start sites of expressed genes that exhibit strong RNA polymerase pausing and that depletion of Gro leads to release of polymerase pausing and increased transcription at a bona fide target gene. Our results demonstrate that Gro is recruited to local sites by transcription factors to attenuate rather than silence gene expression by promoting histone deacetylation and polymerase pausing.

## Author Summary

Repression by transcription factors plays a central role in gene regulation. The Groucho/Transducin-Like Enhancer of split (Gro/TLE) family of co-repressors interacts with many different transcription factors and has many essential roles during animal development. Groucho/TLE proteins form oligomers that are necessary for target gene repression in some contexts. We have profiled the genome-wide recruitment of the founding member of this family, Groucho (from *Drosophila*) to gain insight into how and where it binds with respect to target genes and to identify factors associated with its binding. We find that Groucho binds in discrete peaks, frequently at transcription start sites, and that blocking Groucho from forming oligomers does not significantly change the pattern of Groucho recruitment. Although Groucho acts as a repressor, Groucho binding is enriched in chromatin that is permissive for transcription, and we find that it acts to attenuate rather than completely silence target gene expression. Thus, Groucho does not act as an “on/off” switch on target gene expression, but rather as a “mute” button.

## Introduction

Understanding how transcription factors regulate gene expression is essential for determining how genetically identical cells adopt different fates during animal development. The expression of key genes involved with cell fate determination is often controlled by spatially restricted localization or activity of transcriptional repressors. Many repressors do not have intrinsic repressive activity but recruit co-factors that inhibit productive transcription.

The Groucho/Transducin-Like Enhancer of split (Gro/TLE) family of co-repressors are conserved across metazoa and include a single ortholog in *Drosophila* (Gro), and four orthologs in humans (TLE1-4) and mouse (Gro-related-gene: Grg1-4) (reviewed in [1]–[4]). Gro family proteins do not bind DNA directly, but are recruited to target genes by DNA-binding transcription factors. Gro was first found as a co-factor for Hairy and the related Enhancer of split basic helix loop helix proteins [E(spl)-bHLHs] and Deadpan (Dpn) proteins during neurogenesis, segmentation, and sex differentiation in *Drosophila* [5]. Subsequently, Gro family proteins have been identified as co-repressors for many other transcription factor families including Runx, Nkx, LEF1/Tcf, Pax, Six, Fox and c-Myc (reviewed in [1], [6]). Recruiting partners for Gro/TLE proteins include transcription factors that are effectors of signaling pathways that determine cell fate including Notch and Wnt. Thus, Gro family proteins have roles in a variety of biological processes including osteogenesis, somitogenesis, haematopoesis, and stem cell maintenance and proliferation. Furthermore, human TLE proteins have been implicated in a variety of cancers including breast cancer, leukemia and lymphoma (reviewed in [1], [7]).

The primary structure of Gro/TLE proteins includes five distinguishable regions, of which the most highly conserved are the N-terminal glutamine-rich Q domain and the C-terminal WD-repeat domain [8], [9]. Sequences within the Q domain are predicted to form two coiled-coil motifs that facilitate oligomerization of Gro molecules *in vitro* [9]–[11] and also mediate interactions with some repressors [7], [12], [13]. The WD-repeat domain has been shown by X-ray crystallography to form a β-propeller [14], [15], which binds many different transcription factors, including those containing the conserved “eh1” and WRPW and related peptide motifs [15].

One model for Gro repression is that upon recruitment to a target site by a DNA binding transcription factor, Gro oligomerizes along the DNA and recruits factors that modify chromatin to inhibit transcription from promoters that may be over 1 kb from the initial recruitment site [9], [16]. This model is sometimes referred to as the “spreading model” and is based on the observations that oligomerization via the Q domain is required for Gro family proteins to repress reporter gene transcription in *Drosophila* S2 cells and in overexpression assays in the fly [9], [11], and that Gro interacts with a histone deacetylase (HDAC1, referred to as Rpd3 in *Drosophila*; [17]). Recent support for this model comes from the observations that when a LexA-Hairy fusion protein recruits Gro to a reporter gene in flies, Gro recruitment is spread across 2–3 kb of the gene and is associated with Rpd3 recruitment and reduced histone acetylation [18]. Gro-mediated repression of the *fushi tarazu* (*ftz*) gene by ectopic expression of Hairy induces histone deacetylation for several kilobases around *ftz* [19]. Furthermore, the presence of histone deacetylase inhibitors or decreasing the dose of Rpd3, lessen the defects caused by overexpressing Gro in wing imaginal discs in *Drosophila* [20]. However, Gro repression is only partially dependent on Rpd3, indicating that other modes of repression by Gro are important *in vivo* [20], [21].

Analysis of an endogenous *Drosophila* mutation revealed that oligomerization is not always required for the co-repressor function of Gro. *gro^MB12^* is a single base pair substitution in the translation initiator ATG codon (ATG-ATA) that leads to an N-terminal truncation, deleting much of the Q-domain [3]. MB12 protein does not oligomerize *in vitro* and is expressed at <5% normal levels in early embryos. Nevertheless, *gro^MB12^* is not a null: maternal mutant embryos have intermediate segmentation phenotypes and retain more body mass than the null, indicating that MB12 retains some co-repressor activity. The *gro^MB12^* mutation has differential effects on the expression of target genes *in vivo*. For example, repression of the *tailless* (*tll*) gene by the Capicua-Gro complex is relatively normal in *gro^MB12^* embryos while repression of *snail* by Huckebein-Gro fails. Thus, there are differential requirements for oligomerization via the Q domain during Gro-mediated repression.

In this study we have used chromatin immunoprecipitation followed by high throughput sequencing analysis (ChIP-seq) to profile the genome-wide recruitment of wild-type and non-oligomerizing Gro at high resolution in single cell types using *Drosophila* cell culture. In addition, we have focused on Gro recruitment at a known target locus [*E(spl)mβ-HLH*] to establish a model for Gro function as a co-repressor.

## Results

### Genome-wide profile of Gro recruitment in Kc167 cells

To profile genome-wide Gro binding in Kc167 cells, we performed ChIP-seq using a previously validated anti-Gro antibody [22]. We chose Kc167 cells as they had been characterized extensively for genome-wide transcription factor binding, chromatin modifications and gene expression by Filion et al., [23] and the modENCODE project [24]. Use of a single cell type avoided the complications of interpreting data derived from multiple cell types (e.g. embryo collections) where peaks may represent binding to overlapping or adjacent regulatory elements used at different times or by specific cell types.

**Figure 1:**
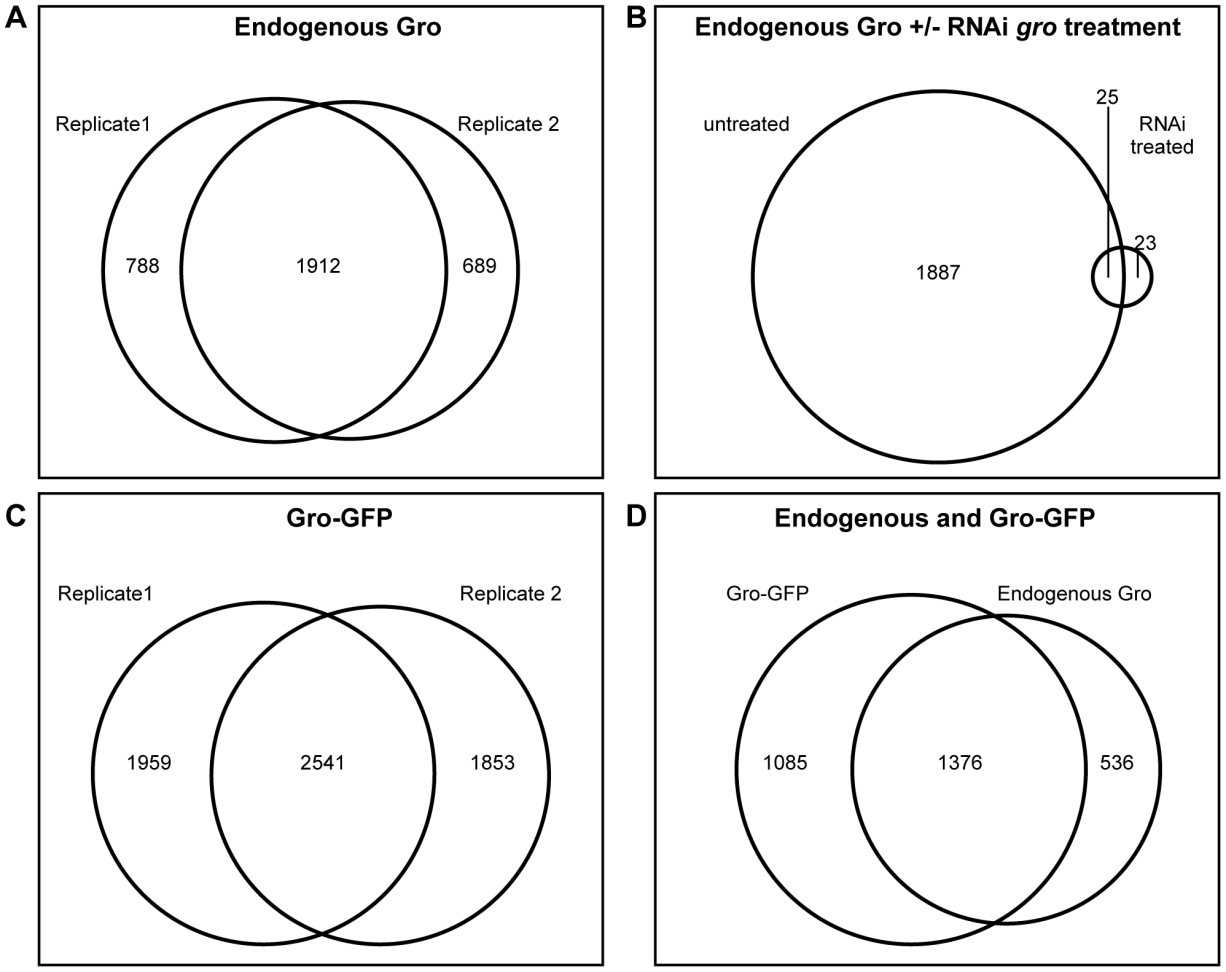
Genome-wide profile of Gro recruitment in Kc167 cells. A) Venn diagram showing the relationship between 2 ChIP-seq biological replicates generated using the anti-Gro antibody. B) Venn diagram illustrating the relationship between ChIP-seq peaks derived from untreated Kc167 cells, and Kc167 cells depleted of Gro by RNAi. C) Venn diagram showing the relationship between 2 ChIP-seq biological replicates generated using the anti-GFP antibody in Kc167 cells transfected with Gro-GFP. D) Venn diagram illustrating the overlap between peaks of endogenous Gro and Gro-GFP recruitment.

Gro binding sites were determined by the maximum per cent overlap of called peaks in two independent biological samples (see Materials and Methods for further details). This analysis yielded 1912 peaks of endogenous Gro binding (Figure 1A). Depletion of Gro from Kc167 cells using RNAi against the 3′-untranslated region of the endogenous *gro* transcript led to a dramatic reduction of the number of significant peaks, demonstrating that ChIP with the anti-Gro antibody reflects bona fide Gro binding (Figure 1B).

As subsequent experiments would require the expression of a mutated variant of Gro, we generated a wild-type Gro tagged with GFP (Gro-GFP), tested its recruitment (using an anti-GFP antibody) in Kc167 cells depleted of endogenous Gro, and compared replicate samples as above (Figure 1C). To compare binding between the endogenous and GFP-tagged Gro, replicate samples were normalized together with the input, and the mean log fold change (FC) for each condition plotted. The results were highly similar to the endogenous Gro (Figure 1D) and we therefore generated a “superset” of high confidence bound regions in Kc167 cells by selecting the 1376 peaks common to all datasets (Table S1).

### Gro binds in discrete peaks across the genome

**Figure 2:**
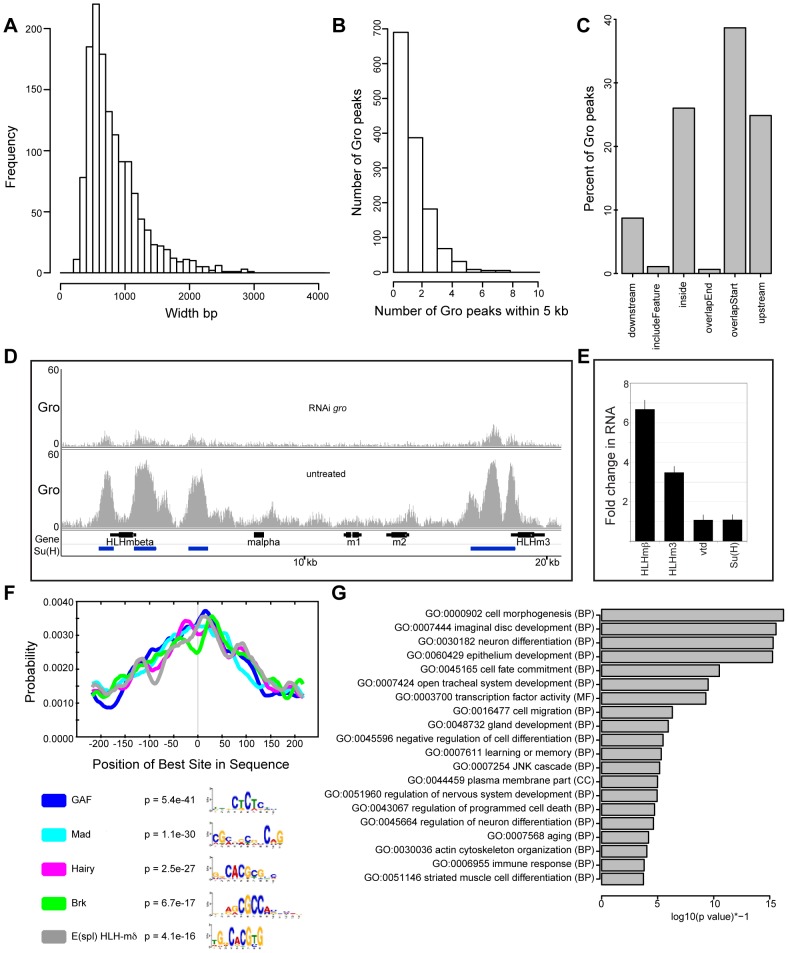
Characterization of high confidence Gro binding sites in Kc167 cells. A) Histogram showing the frequency of peak widths (100 bp bins) of high confidence Gro binding sites in Kc167 cells. B) Histogram showing the number of peaks observed within 5 kb of another Gro peak. C) Plot showing the position of Gro recruitment in Kc167 cells with respect to annotated transcripts. Note: ‘includeFeature’ means the Gro peak covers the entire transcript and ‘inside’ means the peak is within the transcript boundary. D) Pattern of Gro recruitment in the *E(spl)-C* in wild-type and Kc167 cells depleted of Gro by RNAi. Peaks of Su(H) binding from ChIP-chip analysis (FDR ≤1 from [28]) are marked as blue bars under the gene names. E) Plot showing up regulation of *E(spl)mβ-HLH* and *E(spl)m3-HLH* expression in Kc167 cells treated with *gro* RNAi detected by quantitative PCR. *vtd* and *Su(H)* were included as controls. Jinghua Li and Sarah Bray contributed the data for this panel. F) Centrimo analysis of Gro motif binding in Kc167 cells (Bailey and Machanick, 2012). G) Gene Ontology Analysis of genes associated with Gro peaks. Terms were selected by taking the most significant term (p value<10^−4^) in a cluster and the most significant unclustered terms generated from an analysis with DAVID [67].

We first examined the breadth of peaks bound by Gro in Kc167 cells to determine if Gro is recruited to discrete sites or spreads along the DNA - or if both types of recruitment occur but are target dependent. The model that Gro spreads along chromatin (via Q domain oligomerization) to act as a long-range repressor predicts that Gro peaks would be typically greater than 1 kilobase wide and range to several kilobases [6], [9], [16], [18]. Previous studies of genome-wide Gro recruitment have either lacked the resolution to examine this due to the methodology used (DamID; [23]) or because they were performed using a highly mixed population of cells (0–12 hour embryos; [22]). Our superset of high confidence ChIP-seq peaks of Gro in Kc167 cells typically span less than 1 kb (Figure 2A) with a mean width of 831 bp and a median width of 708 bp (Table S1). Less than 3% (36 peaks) of Gro bound regions extend beyond 2 kb, with the largest being 2922 bp (in the region of *Rh5*).

Peaks exclusive to individual replicates of Gro ChIP-seq tended to be narrower than those peaks found in the high confidence superset (Figure S1), indicating that selection of the superset did not exclude broad peaks found in individual replicates. 33% of Gro peaks in the superset overlapped regions of the genome bound by Gro-Dam in Kc167 cells (DamID data from [23]) (Figure S2A). This is comparable to the overlap observed for ChIP-seq and DamID peaks of GAGA factor [GAF; encoded by Trithorax-like (*Trl*)] (Figure S2B). The conditions used during Gro-Dam analysis may have allowed the detection of broader, lower affinity Gro complexes on the chromatin that were potentially disrupted by the sonication regime necessary for Gro and Gro-GFP ChIP-seq. However, the Gro-Dam peaks that did not overlap with peaks in our ChIP-seq replicates tended to be narrower than those which overlapped with Gro ChIP-seq peaks (Figure S3). This indicates that the Gro-GFP ChIP-seq analysis was not biased against detecting broad Gro peaks.

We also compared the profile of Gro peak widths with those of other transcriptional regulators in Kc167 cells for which ChIP-seq data was currently available. Gro peaks were broader than those produced by GAF, but were narrower than Tramtrack (Ttk), Kruppel (Kr), Zn finger homeodomain 1 (Zfh1) and C-terminal Binding Protein (CtBP) ChIP-seq peaks in Kc167 cells (Figure S4). Peaks from Hairy and Suppressor of Hairless [Su(H)], proteins known to recruit Gro, were found over a broad range of sizes up to 5000 bp. More generally, the dimensions we observe for Gro peaks correspond to peak widths observed from ChIP-seq experiments profiling “point sources” rather than “broad sources” [25].

Our data demonstrate that Gro binding is not typically spread over multi-kilobase regions of the genome, while the conditions and analysis we used did not exclude the recovery of ≥2 kb peaks. However, several genomic regions contain clusters of discrete Gro peaks that are spread across several kilobases (Table S1 and Figure 2B,D) that could be interpreted as single broad peaks using techniques and analysis with lower resolution.

Gro peaks commonly overlap annotated transcription start sites in Kc167 cells, although peaks are also found upstream of and inside genes (Figure 2C). One region that contains a cluster of Gro bound sites is the *Enhancer of Split Complex* [*E(spl)-C*] (Figure 2D). Gro has previously been shown to form a complex with Hairless (H) and [Su(H)], contributing to the repression of target genes in the absence of Notch signaling [26], [27]. Su(H) represses Notch target gene expression (including *E(spl)-C* genes) in the absence of Notch signaling in Kc167 cells [28]. We therefore assessed whether there was a relationship between the Gro and Su(H) bound regions within the *E(spl)-C*. The Gro peaks overlapped Su(H) peaks close to *E(spl)mβ-HLH* and *E(spl)m3-HLH* (Figure 2D). The expression of *E(spl)mβ-HLH* and *E(spl)m3-HLH* was increased in Kc167 cells treated with Gro RNAi (Figure 2E, Table S2).

To test if depletion of Gro is sufficient to induce gene expression of repressed targets, we compared gene expression by RNA-seq of untreated and *gro* RNAi Kc167 cells. There were very few genes differentially expressed genes and when looking at the whole transcriptome, we did not observe a general induction of genes (e.g. at below statistical significance) closely associated with ChIP-seq peaks in RNA-seq analysis (Table S2, Figure S5), although the expression of two high confidence target genes within the *E(spl)-C* is upregulated when Gro is depleted by RNAi.

Gro is recruited as a co-factor by many different DNA-binding transcription factors in addition to Su(H), thus Gro peaks are not expected to contain one consensus DNA binding sequence. In agreement with this, no single consensus motif was found in the high confidence Gro peaks (Figure 2F). Instead, binding motifs for several different transcription factors expressed in Kc167 cells [29] with unrelated consensus recognition sequences were enriched in Gro peaks [30]. These included binding motifs for known partners of Gro, including Hairy and Brinker (Brk). In addition, motifs for GAF and Mothers against dpp (Mad), which have not previously been identified as Gro partners, were also enriched in Gro bound regions.

Gene Ontology analysis revealed that the terms over-represented in the genes nearest Gro binding sites in Kc167 cells included “cell morphogenesis”, “imaginal disc development” and “neuron differentiation” (Figure 2G). These terms are consistent with Gro's characterized biological role as a transcriptional co-repressor of developmentally regulated pathways, giving support to our ChIP-seq analysis representing bona fide Gro recruitment.

### Comparison of Gro recruitment in Kc167 and S2 cell lines

**Figure 3:**
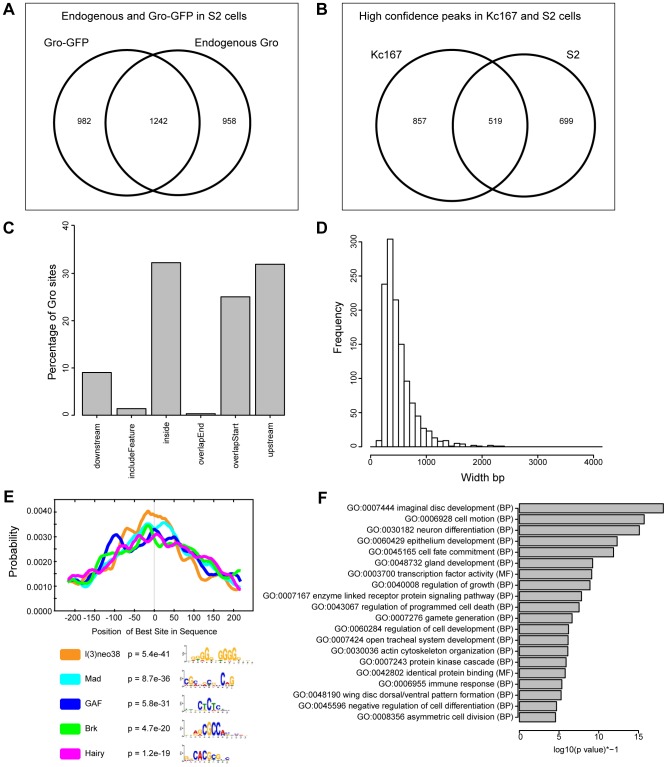
Characterization of Gro recruitment in S2 cells. A) Venn diagram showing the relationship between biological replicates with the most aligned reads from ChIP using anti-Gro and ChIP using anti-GFP (to Gro-GFP) in S2 cells. B) Venn diagram illustrating the overlap of high confidence Gro ChIP peaks in Kc167 and S2 cell lines. C) Plot showing the position of Gro recruitment with respect to annotated transcripts in S2 cells. D) Histogram showing the frequency of peak widths (100 bp bins) of Gro binding sites in S2 cells. E) Centrimo analysis of Gro motif binding in S2 cells (Centrimo; [30]). F) Gene Ontology Analysis of genes associated with Gro peaks in S2 cells. Terms were selected by taking the most significant term (p value<10^−4^) in a cluster and the most significant unclustered terms generated from an analysis with DAVID [67].

To determine if the features of Gro recruitment we observe in Kc167 cells are common to other cell types, we performed ChIP-seq to profile Gro binding in S2 cells. Both Kc167 and S2 cell cultures are derived from late embryonic cells and have properties related to plasmatocytes, but they express distinct profiles of genes [31]. The quality and consistency of the peaks derived from S2 cells were less reproducible between replicates and endogenous versus Gro-GFP ChIP experiments, probably due to the variable aneuploidy observed within S2 cell populations [31]. However, by comparing the replicates with the most reads from ChIP using anti-Gro and ChIP using anti-GFP (to Gro-GFP) we identified 1242 high confidence peaks in S2 cells (Figure 3A, Table S3). 519 of these peaks overlap the superset of high confidence peaks in Kc167 cells (Figure 3B), indicating that the genome-wide profile of Gro recruitment has a cell type specific component. The peaks in S2 cells mapped to a similar profile of genomic features to those in Kc167 cells, although fewer overlapped the start of annotated transcripts (approximately 25% in S2 cells compared to 40% in Kc167; Figure 3C). The high confidence peaks in S2 cells have an average peak width of 503 bp and median width of 425 bp. The widest peak in S2 cells was 2301 bp, and there were just 4 peaks over 2 kb in breadth (Figure 3D). Thus as in Kc167 cells, we did not observe Gro binding over broad domains of the genome in S2 cells.

In common with Kc167 cells, Gro peaks in S2 cells were enriched for GAF, Mad, Brk and Hairy binding sites, but also for l(3)neo38 motifs (Figure 3E). Gene Ontology analysis indicated that the Gro peaks in S2 cells were associated with transcripts linked to developmental processes including “imaginal disc development”, “cell motion”, and “neuron differentiation” (Figure 3F).

We also tested if depletion of Gro is sufficient to induce gene expression of repressed targets in S2 cells. Similar to Kc167 cells, the depletion of Gro from S2 cells by RNAi treatment resulted in very few differentially expressed genes and did not lead to general upregulation of Gro target genes (Table S4, Figure S6).

### Oligomerization of Gro does not contribute to spreading along chromatin

**Figure 4:**
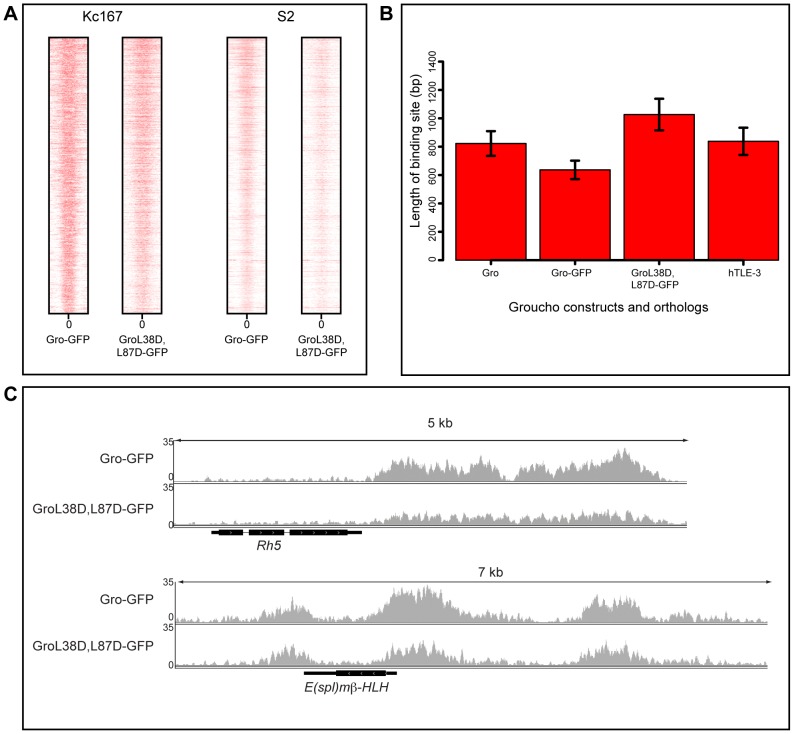
Blocking oligomerization of Gro does not affect peak width in Kc167 or S2 cells. A) Heat maps illustrating the relationship of Gro-GFP and GroL38D,L87D-GFP peaks in Kc167 and S2 cells. Plots extend 500 bp either side of the center of each peak (0) and are ordered by the width of the peak. B) Plot of the average ChIP peak widths obtained for endogenous Gro, Gro-GFP and GroL38D,L87D-GFP in Kc167 cells and for human TLE3 in the MCF7 cell line (TLE3 ChIP-seq data from [32]; GEO accession no. GSM1019137). Error bars represent 95% confidence intervals on the estimates of the means. C) Binding of Gro-GFP and GroL38D,L87D-GFP around the *Rh5* and *E(spl)mβ-HLH* loci in Kc167 cells.

To examine the contribution of oligomerization via the Q-domain to the pattern of Gro recruitment, we used ChIP-seq to compare the binding profiles of a non-oligomerizing variant of Gro tagged with GFP (GroL38D,L87D-GFP; [11]) with Gro-GFP in Kc167 cells depleted of endogenous Gro via RNAi. The positions of the peaks of GroL38D,L87D-GFP showed a high degree of correlation with Gro-GFP peaks (Figure S7). Furthermore, blocking oligomerization of Gro did not decrease the average width of the peaks of Gro recruitment in Kc167 cells (Figure 4A,B). Indeed, the average width of peaks bound by GroL38D,L87D-GFP was slightly higher than endogenous Gro and Gro-GFP (Figure 4B). The width of the broadest Gro peak in Kc167 cells (at the *Rh5* locus) was not affected by blocking oligomerization and peaks bound by GroL38D,L87D-GFP at the *E(spl)mβ-HLH* locus closely resembled those bound by Gro-GFP (Figure 4C). We saw no significant changes in the expression of genes bound by GroL38D,L87D-GFP with respect to those bound by Gro-GFP by RNA-seq analysis (Table S5, Figure S8).

Previous experiments demonstrating that the GroL38D,L87D variant is unable to repress transcription of a reporter gene were performed in S2 cells [11]. Thus we repeated the ChIP-seq experiments comparing recruitment and activity of Gro-GFP and GroL38D,L87D-GFP in S2 cells. The results were largely consistent with those obtained using Kc167 cells. Gro-GFP and GroL38D,L87D-GFP exhibited highly similar binding profiles and peak widths in S2 cells (Figure 4A). Furthermore, as in Kc167 cells, we observed no significant changes in the expression of genes bound by GroL38D,L87D-GFP with respect to those bound by Gro-GFP by RNA-seq analysis in S2 cells (Table S6, Figure S8).

To determine if the pattern of Gro binding in discrete peaks was conserved across evolution, we performed meta-analysis on published ChIP-seq data generated by using an antibody to the human Gro ortholog TLE3 in MCF7 cells [32]. The average peak width for TLE3 was not significantly different to that of Gro in Kc167 cells, indicating that it is recruited in a similar manner to Gro and does not typically spread across broad chromatin domains (Figure 4B).

### Gro peaks are associated with hypoacetylated histones

**Figure 5:**
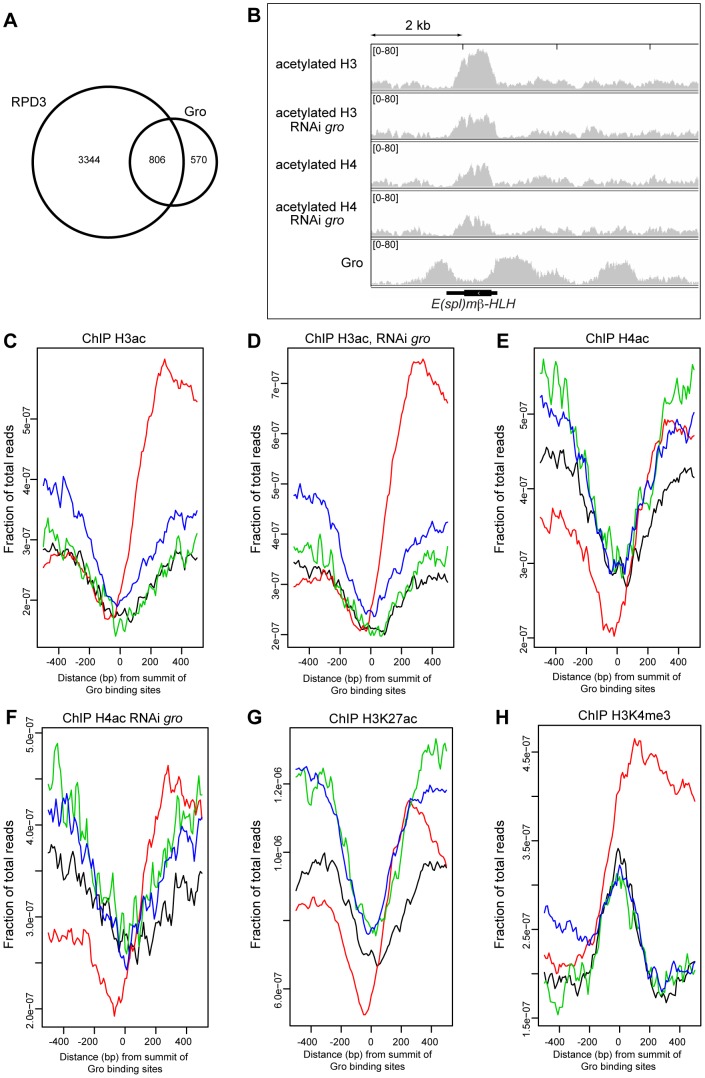
Relationship between Gro recruitment and acetylation status of histones H3 and H4. A) Venn diagram showing the overlap between Gro (superset sites) and HDAC1/Rpd3 binding sites in Kc167 cells. Rpd3 peaks are derived from ChIP-chip data available through modENCODE ([24] http://www.modencode.org). B) Relationship between Gro recruitment and acetylation status of histones H3 and H4 around the *E(spl)mβ-HLH* gene in wild-type and Gro depleted Kc167 cells. C) Average profile of acetylated histone H3 binding with respect to the location of Gro peaks in wild-type Kc167 cells. (location of Gro sites is indicated by red - TSS, black - upstream of a gene, blue - inside a gene, green - downstream of a gene; the profiles are arranged by the strand of the nearest transcript). D) Profile of acetylated histone H3 binding with respect to the location of Gro peaks in Kc167 cells treated with *gro* RNAi. E) Profile of acetylated histone H4 binding with respect to the location of Gro peaks in wild-type Kc167 cells. F) Profile of acetylated histone H4 binding with respect to the location of Gro peaks in Kc167 cells treated with *gro* RNAi. In C)–F) data from a single ChIP-seq replicate is shown. G) Profile of H3K27ac binding with respect to the location of Gro peaks in Kc167. ChIP-seq data for H3K27ac was from [35]. H) Profile of H3K4me3 binding with respect to the location of Gro peaks in Kc167 cells. ChIP-seq data for H3K4me3 was from [35].

Gro has previously been shown to physically and genetically interact with the histone deacetylase Rpd3 in *Drosophila*, although Gro acts independently of Rpd3 in some contexts [17], [18], [20], [21], [33]. Consistent with these observations, we found that 59% of our superset of Gro peaks overlapped with Rpd3 peaks in Kc167 cells (Figure 5A, Rpd3 peaks from modENCODE ChIP-chip data [24]).

Overexpression of Gro correlates with decreased acetylation of histones H3 and H4 around Gro-repressed targets, and phenotypes due to overexpression of Gro in the fly are partially rescued by histone deacetylase inhibitors [18]–[20]. We observed that the peaks in our Gro superset are associated with sites that are depleted of acetylated histones, although histones in the regions adjacent to Gro binding are frequently acetylated (Figure 5B–G). For example, the gene body of *E(spl)mβ-HLH* contains acetylated histones H3 and H4, but the levels are lower at sites where Gro binds around the gene (Figure 5B).

To determine whether Gro induces changes in the acetylation status of histones around Gro target genes we profiled the acetylation status of H3 and H4 in wild-type and Gro depleted Kc167 cells. Knockdown of Gro did not result in any significant changes in H3 or H4 acetylation profiles (Figure 5B–F). There was no significant effect on histone acetylation around the *E(spl)mβ-HLH* gene, which undergoes increased transcription when Gro is depleted (Figures 5B, S9). Thus we found no evidence that depletion of Gro directly influences levels of H3 and H4 acetylation at Gro target sites in Kc167 cells.

Rpd3 has been implicated in the deacetylation of H3K27ac, a chromatin modification that is enriched at active enhancers and promoters in *Drosophila* embryos [34], [35]. Meta-analysis of H3K27ac ChIP-seq data in Kc167 cells [35] reveals that H3K27ac is excluded at Gro peaks (Figure 5G).

The lack of histone acetylation detected at Gro binding sites may have resulted from these regions being nucleosome-free. However, we observe that Gro peaks are enriched for H3K4me3 (H3K4me3 data from [35]), especially when Gro is bound at TSSs (Figure 5H). Promoters are generally marked with high levels of H3K4me3 regardless of their transcriptional state [36]. This overlap indicates that Gro is recruited to sites where there are nucleosomes present that may be modified.

### Gro binding is present in active chromatin and frequently associated with RNAP II at transcription start sites

**Figure 6:**
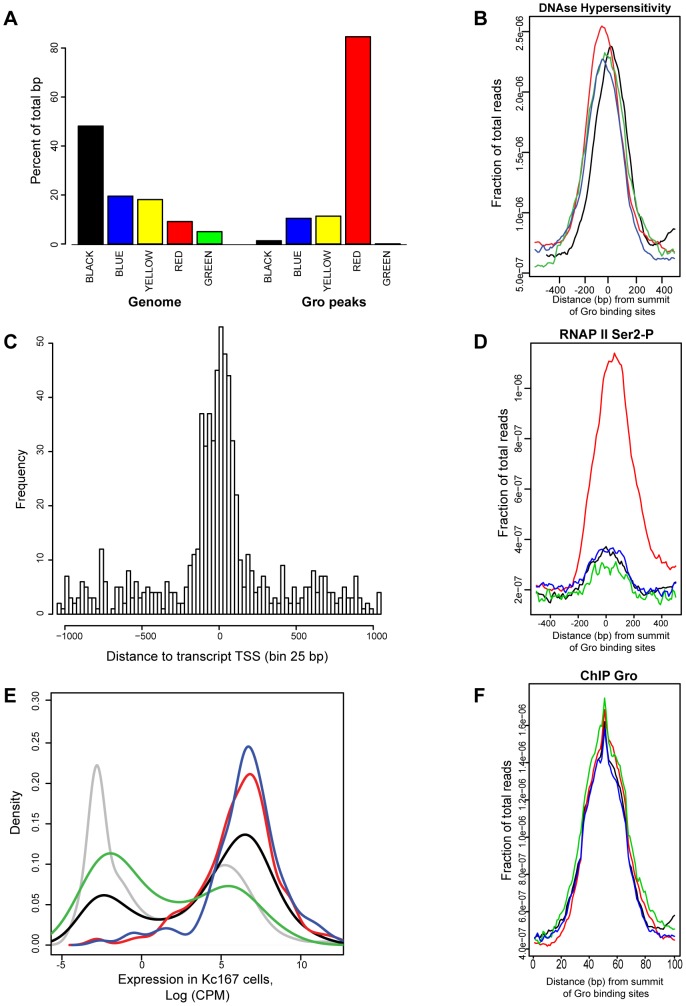
Analysis of the relationship between Gro, chromatin class and RNAP II recruitment in Kc167 cells. A) Enrichment of Gro peaks in the different classes of chromatin defined by [23]. The class of chromatin is indicated by the colour of the bar and in text underneath. The plot is based on the percentage of Gro binding sites (100 bp near the summit of each peak) within each chromatin class. The plot also includes the per cent of the genome based on the number of base pairs that can be mapped to each chromatin class. B) Average profile of DNase hypersensitive sites with respect to the location of Gro peaks in Kc167 cells. The data for DNase hypersensitive sites was obtained from modENCODE [24]. Location of Gro sites is indicated by red - TSS, black - upstream of a gene, blue - inside a gene, green - downstream of a gene; the profiles are arranged by the strand of the nearest transcript). C) Histogram showing where Gro is binding relative to annotated transcript TSSs. The distance is from the summit of the Gro peak to the nearest TSS, adjusted for strand used for transcription. D) Average profile of RNAP II (Ser2-P form) binding at Gro peaks at different locations. E) Density plot showing expression levels of genes with respect to the site of Gro recruitment. red - TSS, black - upstream of a gene, blue - inside a gene, green - downstream of a gene. The expression level of all annotated genes is shown in grey. F) Plot of the average amount of Gro binding at different locations with respect to genes (location of Gro sites is indicated by red - TSS, black - upstream of a gene, blue - inside a gene, green - downstream of a gene).

Integrative analysis of the binding profiles of 53 DamID tagged chromatin associated factors in Kc167 cells produced a model in which the *Drosophila* genome contains five principal chromatin types [23]; “Red” (active, developmentally regulated), “Yellow” (active, housekeeping), “Blue” (repressed, by Polycomb Group complexes) “Green” (repressed, classic heterochromatin), and “Black” (highly repressed). In agreement with [23] (who used Gro-DamID to map Gro binding), we found Gro ChIP-seq peaks were most highly enriched in Red chromatin (Figure 6A), which is associated with factors linked to active, developmentally regulated gene expression. Gro binding appears to be excluded to some extent from the Black and Green types of repressed chromatin. Furthermore, Gro peaks were found in regions associated with DNase I hypersensitivity (Figure 6B), indicating that they lie in open chromatin where the turnover rate of nucleosomes is high [37].

Although Gro may act as a “long range” repressor over distances of greater than 1 kb from the target promoter (reviewed in [38]), we found that almost 40% of Gro peaks overlapped with transcription start sites (TSSs) in Kc167 cells (Figure 2C). Indeed, high resolution mapping revealed that the summits of Gro peaks most frequently map immediately downstream (25–50 bp) of the TSS (Figure 6C) suggesting that Gro often acts on TSSs from a very short range. However, the level of recruitment of Gro to different locations around genes was comparable (Figure 6F).

Since Gro primarily bound annotated TSSs in Kc167 cells, one potential mechanism through which Gro could mediate repression would be to block RNAP II recruitment to TSSs. We used ChIP-seq to profile RNAP II binding to determine if RNAP II is excluded from TSSs bound by Gro. We found that the majority of Gro peaks found at TSSs overlap RNAP II peaks in Kc167 cells, indicating that Gro does not mediate repression by simply blocking RNAP II recruitment (Figure 6D). We observed that peaks of Gro binding that were not localized to TSSs did not show an association with RNAP II recruitment (Figure 6D). We detected transcripts in RNA-seq experiments from genes where Gro was bound at either the TSS or inside the gene (Figure 6E) indicating that these genes were not completely silenced.

### Gro is enriched at transcription start sites that exhibit RNAP II pausing

Since Gro binding at TSSs does not exclude RNAP II recruitment, we attempted to establish if Gro affected the productivity of RNAP II. One way Gro could attenuate transcription would be to promote promoter proximal RNAP II pausing (reviewed in [39]–[42]). Regulation of RNAP II release at the early elongation checkpoint is a major form of transcriptional regulation at genes directing anterior-posterior (AP) and dorsal-ventral (DV) patterning in the early *Drosophila* embryo, which include many known targets of Gro repression [42]–[44].

**Figure 7:**
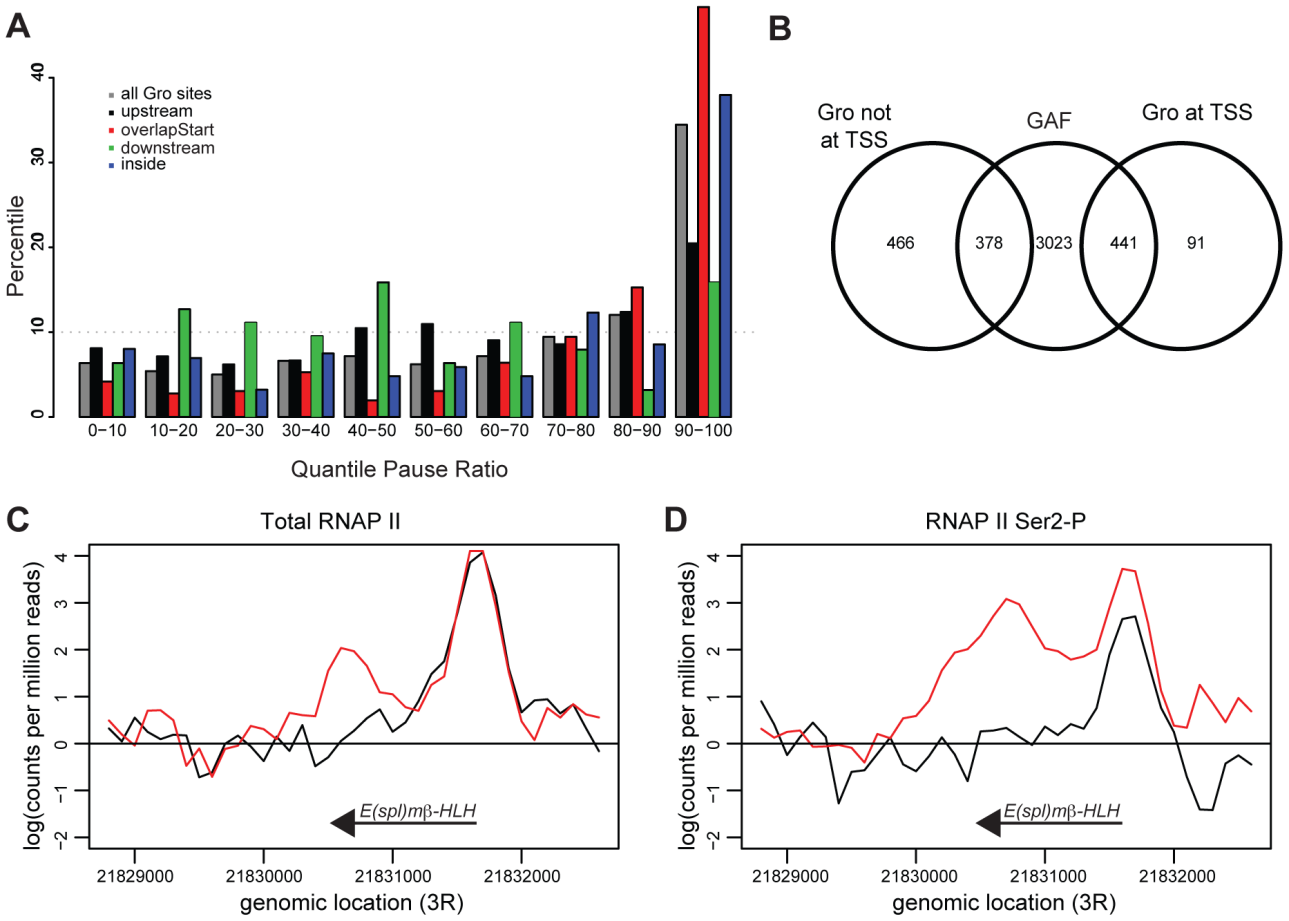
Gro is enriched at genes that exhibit RNAP II promoter proximal pausing. A) Plot showing binding of Gro with respect to polymerase pausing. The pause ratio for RNAP II at annotated TSSs was calculated and divided into 10 quantiles (0–10% has lowest 10% of paused ratio, 90–100% has highest 10% of paused ratio). The percentages of transcripts nearest to Gro binding sites that fall into each quantile were calculated. B) Venn diagram illustrating the overlap between Gro and GAGA Factor (GAF) binding. The GAF peaks were derived from ChIP-seq data generated as part of the modENCODE project ([24]; http://www.modencode.org). C) Profile plot of total RNAP II (using anti-Rbp3 antibody) and D) elongation competent RNAP II (using anti-Ser2-P antibody) across the *E(spl)mβ-HLH* locus in Kc167 in untreated (black) and *gro* RNAi treated (red) cells. Profiles were taken from the average normalized counts of 100 bp fragments from an analysis in edgeR [68].

To determine if Gro peaks were enriched at the start of transcripts that exhibit RNAP II pausing, the pause ratio of all transcripts was determined by establishing the ratio of total RNAP II at the TSS to that within the gene body. Almost 50% of transcripts where Gro is bound at the TSS had a very high pause ratio (in the top 10% of all transcripts; Figures 7A, S10). Furthermore, 82% of Gro peaks located at TSSs overlapped peaks of GAF binding (Figure 7B). GAF has previously been linked to promoter proximal pausing at many genes in *Drosophila* [45], [46]. The analysis therefore suggests that Gro is enriched at TSSs where there is promoter proximal pausing of RNAP II. We did not detect any significant global effects on RNAP II pausing in cells depleted of Gro by RNAi. However, we observed decreased RNAP II pausing at the *E(spl)mβ-HLH* locus, which is a high confidence target of Gro repression in Kc167 cells (Figure 7C,D).

## Discussion

Gro was first described as a “long-range” co-repressor that could inhibit transcriptional initiation of reporter genes while bound to a distant (>1 kb away) enhancer element [47]. However, the model that Gro spreads over multi-kilobase domains to repress transcription was derived from experimental approaches that lacked the resolution to determine if Gro was bound in continuous or clustered peaks around genes. For example, Martinez and Arnosti [18] used ChIP and subsequent qPCR at sites spaced ≥1 kb apart around their single target gene to test the spreading model. The Gro detected at the promoter and at 1 kb, 2 kb and 4 kb upstream of their target gene may have been derived from distinct, discrete peaks of Gro binding. We observe that clusters of Gro peaks across the genome are common (Figure 2B). One example of this occurs at the *E(spl)mβ-HLH* locus where distinct Gro peaks lie less than 2 kb apart, either side of the coding region (Figure 2D). It seems most likely that these are distinct peaks, as they lie over distinct Su(H) peaks and are separated by peaks of histone H3 and H4 acetylation (Figures 5B, S7).

By selecting our superset of high confidence peaks common to all datasets for endogenous Gro and Gro-GFP, we may have excluded some “real” peaks from our general analysis. However, the properties of the peaks excluded from the superset did not differ significantly from the peaks in the superset. In general, peaks that were unique to one replicate were narrower than those included in the superset, further supporting the argument that our conditions and analyses were not biased against recovering broad peaks (Figure S1).

33% of our high confidence Gro ChIP-seq peaks overlapped previously published Gro DamID peaks. This overlap is relatively low, however, a comparable level of overlap (34%) is observed between GAF ChIP-seq and GAF DamID peaks (Figure S2). The Dam domain was fused to the C-terminal domain of Gro [48], which is highly structured and interacts with many classes of transcription factor [15]. Thus, the fusion of the Dam domain to the C-terminal of Gro may have interfered with Gro recruitment to the genome and excluded sites that we could detect with ChIP-seq.

Consistent with Martinez and Arnosti [18], we were unable to obtain reproducible ChIP samples for Gro without the use of a two-step crosslinking method. This may reflect that Gro is not directly recruited to chromatin, but rather via intermediate sequence specific DNA binding transcription factors. Use of two cross-linking agents meant that relatively long sonication was required to generate DNA fragments of a suitable size for sequencing (Materials and Methods). Extended sonication may disrupt indirect chromatin interactions and select only for high affinity binding sites [49]. However we recovered peaks with widths up to 2.9 kb from Kc167 cells (Table S1, Figure 4C) indicating that the sonication regime was not inhibiting the recovery of broad peaks per se. Furthermore, previously published Gro-Dam peaks that overlapped our ChIP-seq peaks tended to be broader than those that did not (Figure S3), indicating that our analysis was not biased against detecting any broad low affinity Gro peaks.

While we do observe some peaks of Gro binding in intergenic regions that may be associated with enhancer elements that are more than 1 kb from the nearest annotated TSS, our data support a model in which Gro is recruited locally by transcription factors and does not spread along the chromatin by oligomerization when it acts on a distant target promoter. Thus, it is most likely that Gro recruited to distant regulatory elements is brought into the proximity of target promoters by “looping” of the DNA. It is well established that chromatin looping can facilitate gene activation by bringing factors bound at intergenic enhancers into contact with the transcription machinery [50], [51] and also facilitate repression by distant regulatory elements [52]. Future studies using chromatin capture techniques in wild-type and Gro depleted cells will determine if Gro contributes to the formation and stability of chromatin loops from distant *cis-*regulatory elements to target promoters.

The RNA-seq experiments did not reveal a general upregulation of genes closely associated with Gro ChIP-seq peaks in cells treated with *gro RNAi* in either Kc167 or S2 cells (Figures S5, S6, Tables S2, S4). Indeed treatment with *gro* RNAi led to very few significant changes in gene expression. Similarly, we did not observe widespread Gro-related changes to histone acetylation status or RNAP II recruitment or pausing. We only observed highly significant changes to gene expression and RNAP II recruitment at a single known Gro target, *E(spl)mβ-HLH*. It is possible that loss of Gro may have led to increased variability in target gene expression, and the average expression values from many cells in our two biological replicates is unlikely to be sufficient to show any change in variability. However, genome-wide loss of Gro from its targets may not facilitate recruitment of activating factors in the absence of other changes in the nuclear environment (e.g *de novo* expression of transcription factors in response to cell-cell signaling). In addition, the residual Gro in these cells may be sufficient to maintain repression of most target genes (Figure S5, S7C). The use of *gro^null^* cells made by newly available genome engineering techniques [53] may resolve this in the future - if *gro^null^* cells are viable.

Previous overexpression studies in S2 cells and in the fly indicate that oligomerization affects how Gro acts in cells [9], [11]. For example, ectopic expression of wild-type Gro leads to ectopic repression of the *vgQ-lacZ* reporter gene whereas overexpression of the non-oligomerizing GroL38D,L87D variant has no detectable effect on *vgQ-lacZ* expression [11]. We do not observe dramatic differences in the breadth or location of Gro peaks with a variant that does not oligomerize (L38D,L87D-GFP), lending support to the alternative models that it is the efficiency of Gro recruitment or overall structure of the co-repressor complex that is compromised in the presence of non-oligomerizing variants [9]. We observe an apparent reduction in the amount of L38D,L87D-GFP binding with respect to Gro-GFP at the *Rh5* locus (Figure 4C) although this effect is not observed at *E(spl)mβ-HLH*. This indicates that the level of Gro binding may be dependent on oligomerization at a subset of targets. Genetic evidence indicates that gro is not expressed in vast surplus to requirement as many genetic interactions can be detected with *gro* heterozygotes. For example, multiple *gro* mutations were isolated in screens for dominant suppressors of *ro^Dom^* [54] and ectopic Hairy expression in the eye [55].

Our results are generally consistent with those from previous studies that identified an association of Gro with hypoacetylated histones H3 and H4 [17], [20], [21]. However, we did not detect significant changes in the histone acetylation status of histones H3 and H4 at Gro target sites when we reduced Gro levels in Kc167 cells. We cannot formally rule out that the residual Gro left in cells treated with RNAi against *gro* is sufficient to maintain histones in a hypoacetylated state or that there are subtle changes to acetylation levels that cannot be accurately detected by ChIP-seq methods. Furthermore, loss of repression and gene activation are separable processes and depletion of Gro did not facilitate the recruitment and activity of histone acetylases at levels that we could detect.

Recent studies have revealed that regulation of promoter proximal pausing by RNAP II is a major point of control of the expression of many genes that respond to developmental and environmental cues. Paused polymerase is highly enriched at genes in stimulus-responsive pathways [56] and in genes involved with patterning the axes in the early *Drosophila* embryo [44]. Strikingly, Gro has critical functions regulating gene expression in stimulus-responsive pathways (e.g. Notch and Wnt signaling) and both AP and DV patterning. It has been proposed that pausing contributes to the plasticity of gene expression by keeping genes that must be repressed transiently in a state permissive for rapid reactivation [44], [56], [57]. Gro-mediated repression is frequently dynamic and rapidly reversible during animal development. For example, the serial production of *Drosophila* embryonic neuroblasts relies on five short pulses of Notch signaling that occur within 4 hours [5], [58], [59]. Activation of primary Notch target genes repressed by the Su(H)/Gro complex occurs within 5 minutes of triggering the Notch pathway in *Drosophila* DmD8 cells, and this activation is correlated with reduced RNAP II pausing [60]. We have demonstrated that Gro peaks frequently overlap with peaks of a known regulator of RNAP II pausing (GAF) and that Gro is required to maintain RNAP II pausing at *E(spl)mβ-HLH*, a gene known to be a target of Gro repression via recruitment by Su(H) in Kc167 cells. Although much is known about the molecular mechanisms that control the P-TEFb checkpoint and RNAP II pausing, very little is known about which contextual factors determine the extent of RNAP II pausing. Future studies will address whether Gro interacts with known regulators of the P-TEFb checkpoint to promote RNAP II pausing in a gene-specific manner.

Finally, the finding that Gro target genes are transcribed is consistent with several other genome-wide studies that show association of repressors with actively transcribed loci [61]. It is thought that this class of repressor allows cells to make rapid responses to developmental and environmental cues and to fine-tune levels of active gene expression. Our data indicates that Gro belongs to this class and behaves like a modulator rather than an off switch at its target genes. This work adds to the growing body of evidence that fine-tuning of gene expression is a general mechanism of co-repressor function [61].

## Materials and Methods

### Plasmids and RNA

Gro and GroL38D;L87D cDNA was generated by PCR from cDNA templates PCR4-TOPO-Gro [15] and pRM-GroL38D;L87D ([11], a gift from Alfred Courey). These were cloned into the N-terminal GFP-tagged vector pAGW [*Drosophila* Genomics Resource Centre (DGRC) T. Murphy, unpublished]. Double-stranded RNA against *gro* was generated using the Megascript T7 kit following manufacturer's instructions (Life Technologies) and BAC13F13 (Children's Hospital Oakland Research Institute) as the template following the approach of [11]. The dsRNA was designed to target the *gro* 3′-UTR so that only transcripts from the endogenous *gro* gene were targeted for destruction. The following primers were directed against Gro 3′ UTR (from 95 bp to 683 bp downstream of stop codon) with the additional T7 recognition sequence underlined. Forward: 5′-TAATACGACTCACTATAGG CAACAGCAGCAGCATCGGCAG-3′. Reverse: 5′- TAATACGACTCACTATAGG TGGAGGGACGTTGGGAGGTAAG-3′.

### Cell culture and transfection

Kc167 and S2R+ cells were obtained from the *Drosophila* Genomics Resource Center (DGRC). Transfections were performed using Effectene according to the manufacturer's instructions (Qiagen). Successful transfection and knockdown were assessed by western blot (see Protocol S1).

### Chromatin Immunoprecipitation (ChIP) and sequencing

A more detailed description of the ChIP procedure is provided in Protocol S1. For ChIP using anti-Gro or anti-GFP antibodies, cells were double crosslinked by treatment for 20 minutes at room temperature with Disuccininmidyl glutarate (DSG-Fisher Scientific) followed by formaldehyde treatment. For all other antibodies, samples were single crosslinked by treatment with formaldehyde.

For all Gro and GFP samples at least 2.9 million uniquely aligned reads were generated per replicate and for all other samples at least 7 million reads were generated per replicate. These are above the minimum number of reads recommended by modENCODE project guidelines for *Drosophila* [62].

### ChIP-seq analysis

Illumina MiSeq paired-end and single-end reads were aligned to genome (BDGP 5.70) with Bowtie version 2.1.0 [63] using the alignment parameter set to ‘very sensitive’. Aligned reads were sorted and duplicate reads and reads that did not map uniquely to the genome were removed with samtools version 1.4 [64]. Binding peaks were identified against input samples using MACS version 2 [65] with MFOLD parameters set to 2 and 10.

To identify binding sites present in two biological replicate samples (or between conditions), a large number of peaks were identified in each sample and peaks were ranked by *p* values generated in MACS. The per cent overlap was determined between samples at various ranks and the point of maximum per cent overlap was used as a cutoff to generate a list of peaks present in both samples. Typically, the majority of binding sites had a FDR less than 10%.

ChIPpeakAnno version 2.10.0 [66] was used to annotate binding sites relative to a genomic feature (e.g. nearby gene, TSS or chromatin type) and to identify functional annotation terms that were enriched in the list of nearby genes, we used the Database for Annotation, Visualization and Integrated Discovery (DAVID) v6.7 [67].

To compare the level of binding at particular genomic locations, Rsamtools in R/Bioconductor was used to count reads at 100 bp intervals across the genome. edgeR was used to normalize and identify significant differences between samples [68]. Normalization was performed with upper-quantile method and percentile set to 0.95 so that log2 fold enrichment at the summit of the binding site roughly matched the log2 fold enrichment called by the MACS program.

Centrimo version 4.9.1 [30] was used to identify sequence motifs that were enriched in 500 bp sequences that were centred on the binding peak summit as identified by MACS. The binding motifs were established as follows; GAF, Brk [69], Mad, Hairy [70], E(spl)mβ-HLH, l(3)neo38 [71].

Pause ratios were calculated by HOMER (Hypergeometric Optimization of Motif EnRichment; [72]) using counts from the TSS to 250 bp downstream and counts in the gene body.

### RNA-seq

Total RNA was obtained using the Qiagen RNeasy mini kit. mRNA was then extracted using the Dynabeads mRNA Purification Kit (Life Technologies). mRNA libraries were generated following the manufacturer's instructions (NEBnext mRNA Library Prep Master Mix – E6110S). Samples were sequenced on the Illumina MiSeq following the manufacturer's protocol and paired-end 36 bp reads generated. For all samples two biological replicates were sequenced, and at least 7 million reads generated per replicate.

### RNA-seq analysis

Illumina paired-end reads were aligned to genome (BDGP 5.70) with Bowtie version 2.1.0 [63] and splice junctions were mapped with Tophat version 2.0.8b [73]. edgeR version 3.4.0 (using the default parameters) was used to normalize and identify differentially expressed genes [68]. For identification of over-represented terms in the list of genes differentially expressed we used DAVID v6.7 [67]. P-values were adjusted for multiple testing by the Benjamini & Hochberg (BH) step-up FDR-controlling procedure [74].

### Accession numbers

The accession number for the Illumina Sequencing data from this study on ArrayExpress is E-MTAB-2316.

## Supporting Information

Figure S1 Comparison of peak widths in individual endogenous Gro ChIP-seq replicates. A) Density plot showing peak widths obtained from replicate 1 of endogenous Gro ChIP-seq analysis. The peak widths of subsets of this replicate are shown as indicated. B) Density plot showing peak widths obtained from replicate 2 of endogenous Gro ChIP-seq analysis. The peak widths of subsets of this replicate are shown as indicated.
(PDF)

Figure S2 Overlap between ChIP-seq peaks and DamID peaks in Kc167 cells. A) Venn diagram illustrating the overlap between the high confidence superset of Gro ChIP-seq peaks (Table S1) and peaks obtained using Gro-DamID [23] in Kc167 cells. B) Venn diagram illustrating the overlap between peaks obtained by ChIP-seq to GAF (GEO accession number GSM1318358) and peaks obtained using GAF-DamID [23] in Kc167 cells.
(PDF)

Figure S3 Comparison of peak widths obtained with Gro-DamID with replicates of endogenous and Gro-GFP ChIP-seq. Density plot showing peak widths obtained from Gro-DamID analysis [23] and the widths of subsets of these peaks as indicated.
(PDF)

Figure S4 Comparison of ChIP-seq peak widths obtained for transcriptional regulators in Kc167 cells. Density plot showing peak widths obtained via ChIP-seq for various transcriptional regulators in Kc167 cells as indicated. All data is from the modENCODE project (www.modencode.org) excluding the Gro peaks (the superset of high confidence peaks from this study) and cMyc (accession number GSM970847 on GEO at NCBI).
(PDF)

Figure S5 Gene expression profiles of untreated and *gro* RNAi treated Kc167 cells. A) Plot illustrating the log fold changes (logFC) for all expressed genes (grey) and with genes mapping nearest to a Gro binding site (red). B) Density plot illustrating the distribution of log fold changes (logFC) for all expressed genes and genes nearest to a Gro binding site.
(PDF)

Figure S6 Gene expression profiles of untreated and *gro* RNAi treated S2 cells. A) Plot illustrating the log fold changes (logFC) for all expressed genes (grey) and with genes mapping nearest to a Gro binding site (red). B) Density plot illustrating the distribution of log fold changes (logFC) for all expressed genes and genes nearest to a Gro binding site.
(PDF)

Figure S7 Characterization of Gro-GFP and GroL38D,L87D-GFP recruitment and expression in Kc167 cells. A) Venn diagram showing the overlap between Gro-GFP and GroL38D,L87D-GFP peaks in Kc167 cells. B) Plot showing the log fold change (FC) of Gro-GFP and GroL38D,L87D-GFP peaks (100 bp fragment nearest the summit of each peak) in Kc167 cells after normalization in edgeR [68]. C) Western blot analysis showing the expression of endogenous Gro and GFP-tagged Gro variants (detected by anti-GFP antibody) in untreated and treated Kc167 cells as indicated, with beta-Tubulin included as a loading control.
(PDF)

Figure S8 Comparison of gene expression in cells expressing Gro-GFP and L38D,L87D-GFP by RNA-seq analysis. A) Plot illustrating the log fold changes (logFC) for all expressed genes (grey), genes mapping nearest to a Gro binding site (red) and genes mapping nearest to peaks bound by L38D,L87D-GFP (blue) in Kc167 cells. B) Density plot illustrating the distribution of log fold changes (logFC) for all expressed genes (grey), genes nearest to a Gro binding site (red) and genes nearest L38D,L87D-GFP peaks (blue) in Kc167 cells. C) Plot illustrating the log fold changes (logFC) for all expressed genes (grey), genes mapping nearest to a Gro binding site (red) and genes mapping nearest to peaks bound by L38D,L87D-GFP (blue) in S2 cells. D) Density plot illustrating the distribution of log fold changes (logFC) for all expressed genes (grey), genes nearest to a Gro binding site (red) and genes nearest L38D,L87D-GFP peaks (blue) in S2 cells.
(PDF)

Figure S9 Profiles of Histone H3 and H4 acetylation at the *E(spl)mβ-HLH* locus in Kc167 cells. A) Plot of average level of H3 acetylation across the *E(spl)mβ-HLH* locus from untreated cells (black) and cells treated with *gro* RNAi (red). Profiles were taken from the average normalized counts of 100 bp fragments from an analysis in edgeR [68]. B) Plot of the individual replicate samples used to make the plots in A (after normalization). C) Regions that are significantly different between untreated and *gro* RNAi samples for histone H3 acetylation. Note: this shows −log10 (p value) peaks. D) Normalized plot of H4 acetylation across the *E(spl)mβ-HLH locus* from untreated cells (black) and cells treated with *gro* RNAi (red). E) Plot of the individual replicate samples used to make the plot in D (after normalization). F) Regions that are significantly different between untreated and *gro* RNAi samples for histone H4 acetylation. Note: this shows -log10 (p value) peaks.
(PDF)

Figure S10 Gro is enriched at paused transcripts. Density plot showing the pause ratio of all transcripts (black) and transcripts associated with Gro binding (red).
(PDF)

Protocol S1 Additional details of the materials and methods for ChIP-seq experiments.
(DOCX)

Table S1 High confidence Gro ChIP-seq peaks identified in Kc167 cells.
(XLS)

Table S2 Comparison of gene expression by RNA-seq analysis of untreated and *gro* RNAi treated Kc167 cells.
(XLSX)

Table S3 High confidence Gro ChIP-seq peaks identified in S2R+ cells.
(XLS)

Table S4 Comparison of gene expression by RNA-seq analysis of untreated and *gro* RNAi treated S2 cells.
(XLSX)

Table S5 Comparison of gene expression via RNA-seq analysis of Kc167 cells expressing Gro-GFP and L38D,L87D-GFP.
(XLSX)

Table S6 Comparison of gene expression via RNA-seq analysis of S2 cells expressing Gro-GFP and L38D,L87D-GFP.
(XLSX)
