# Supplementary figures and images for: The Groucho Co-repressor Is Primarily Recruited to Local Target Sites in Active Chromatin to Attenuate Transcription
Source: PLoS Genet. 2014 Aug 28;10(8):e1004595. doi: 10.1371/journal.pgen.1004595 (PMC4148212; doi:10.1371/journal.pgen.1004595)

**A**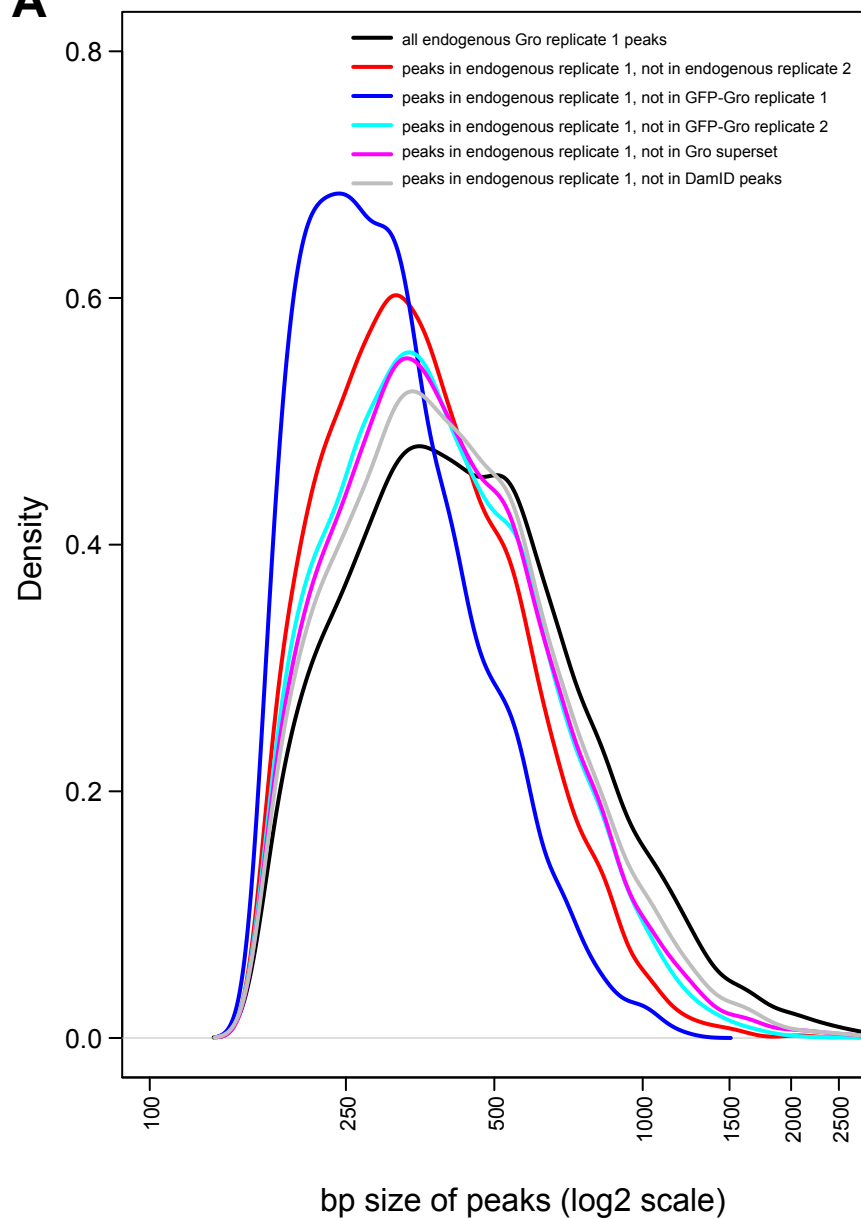**B**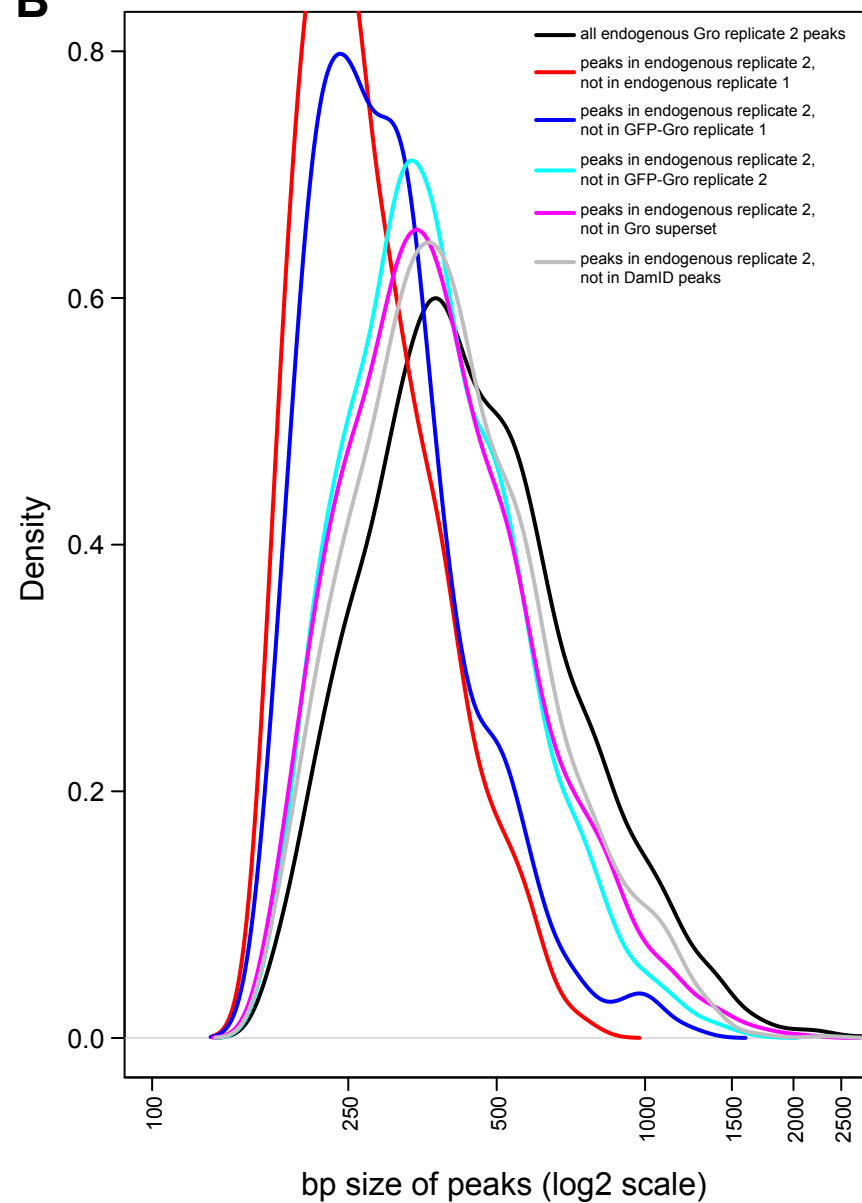

Supplement: Figure S1 — Comparison of peak widths in individual endogenous Gro ChIP-seq replicates. A) Density plot showing peak widths obtained from replicate 1 of endogenous Gro ChIP-seq analysis. The peak widths of subsets of this replicate are shown as indicated. B) Density plot showing peak widths obtained from replicate 2 of endogenous Gro ChIP-seq analysis. The peak widths of subsets of this replicate are shown as indicated. (PDF) [file pgen.1004595.s001.pdf]

**A**

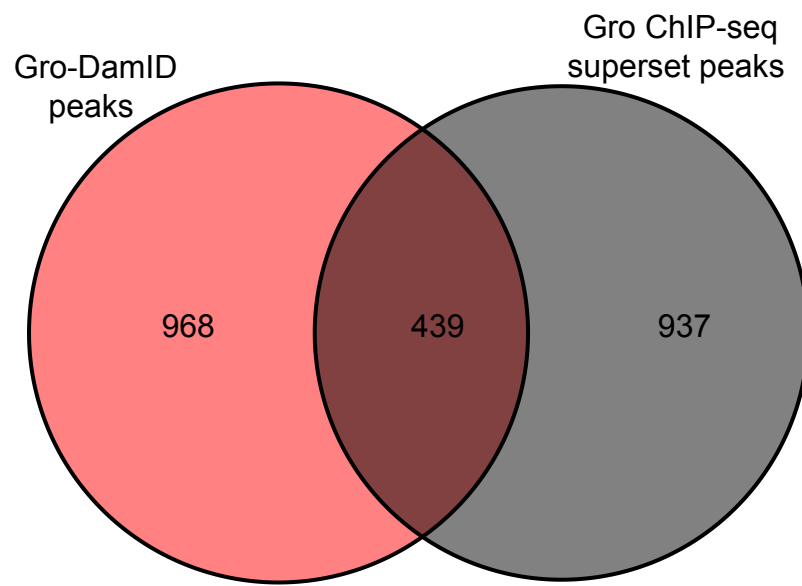

**B**

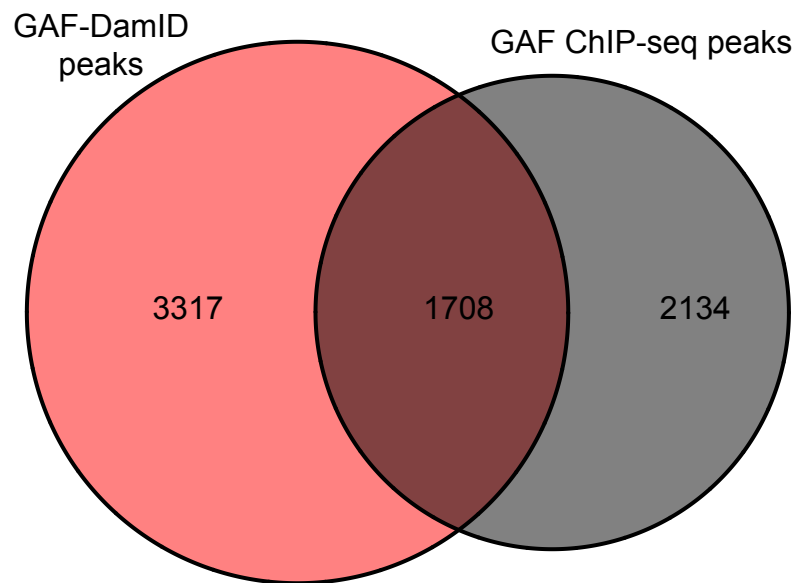

Supplement: Figure S2 — Overlap between ChIP-seq peaks and DamID peaks in Kc167 cells. A) Venn diagram illustrating the overlap between the high confidence superset of Gro ChIP-seq peaks (Table S1) and peaks obtained using Gro-DamID [23] in Kc167 cells. B) Venn diagram illustrating the overlap between peaks obtained by ChIP-seq to GAF (GEO accession number GSM1318358) and peaks obtained using GAF-DamID [23] in Kc167 cells. (PDF) [file pgen.1004595.s002.pdf]

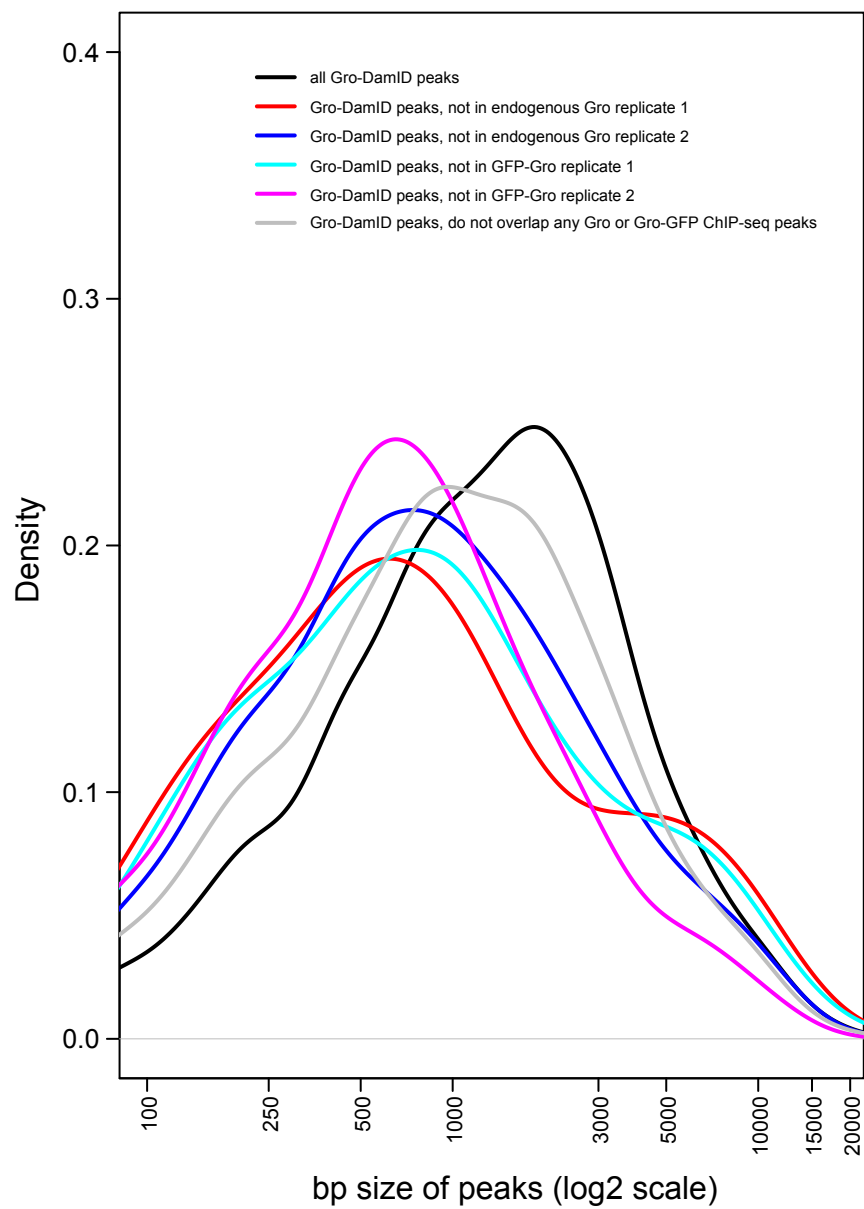

Supplement: Figure S3 — Comparison of peak widths obtained with Gro-DamID with replicates of endogenous and Gro-GFP ChIP-seq. Density plot showing peak widths obtained from Gro-DamID analysis [23] and the widths of subsets of these peaks as indicated. (PDF) [file pgen.1004595.s003.pdf]

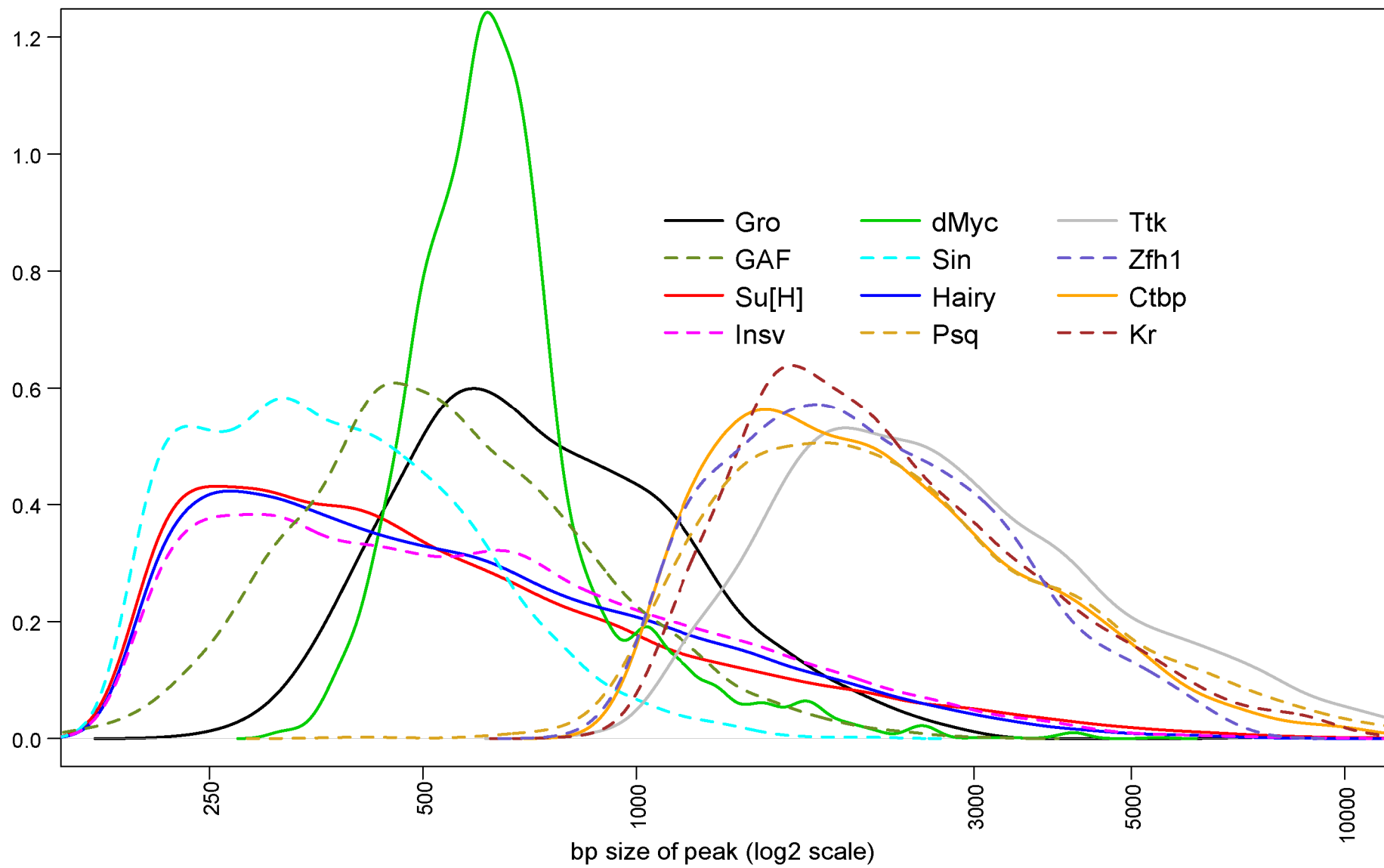

Supplement: Figure S4 — Comparison of ChIP-seq peak widths obtained for transcriptional regulators in Kc167 cells. Density plot showing peak widths obtained via ChIP-seq for various transcriptional regulators in Kc167 cells as indicated. All data is from the modENCODE project (www.modencode.org) excluding the Gro peaks (the superset of high confidence peaks from this study) and cMyc (accession number GSM970847 on GEO at NCBI). (PDF) [file pgen.1004595.s004.pdf]

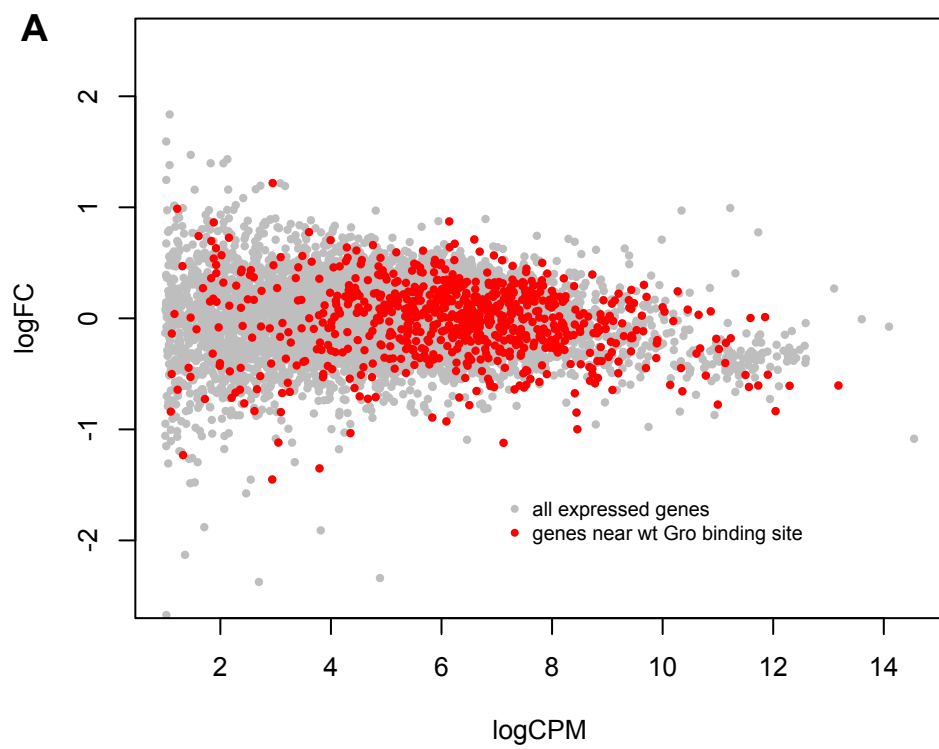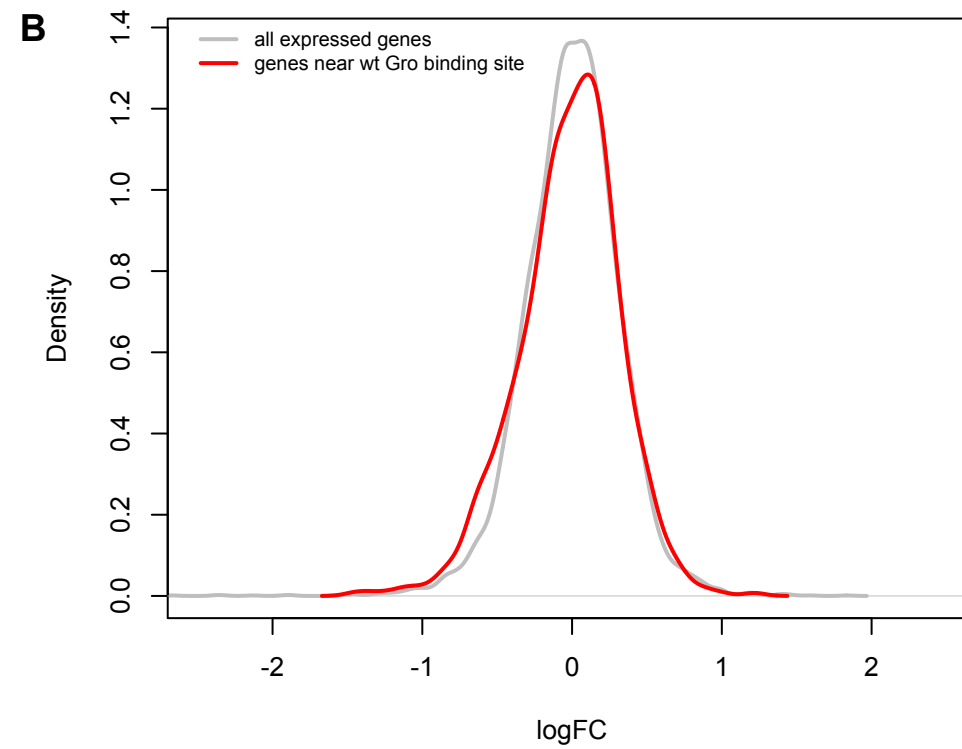

Supplement: Figure S5 — Gene expression profiles of untreated and gro RNAi treated Kc167 cells. A) Plot illustrating the log fold changes (logFC) for all expressed genes (grey) and with genes mapping nearest to a Gro binding site (red). B) Density plot illustrating the distribution of log fold changes (logFC) for all expressed genes and genes nearest to a Gro binding site. (PDF) [file pgen.1004595.s005.pdf]

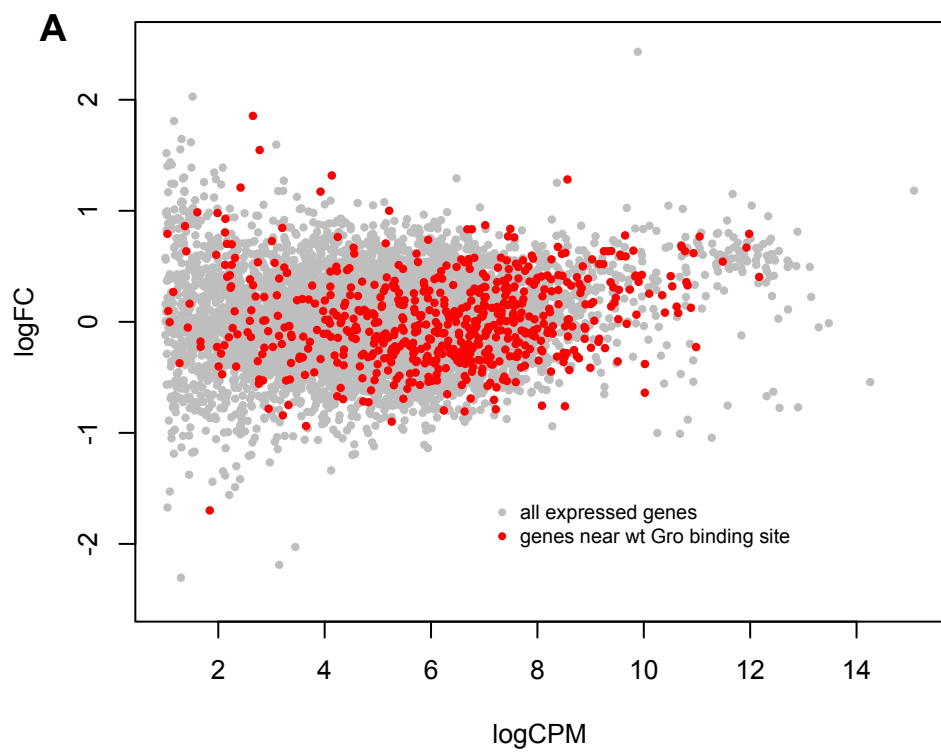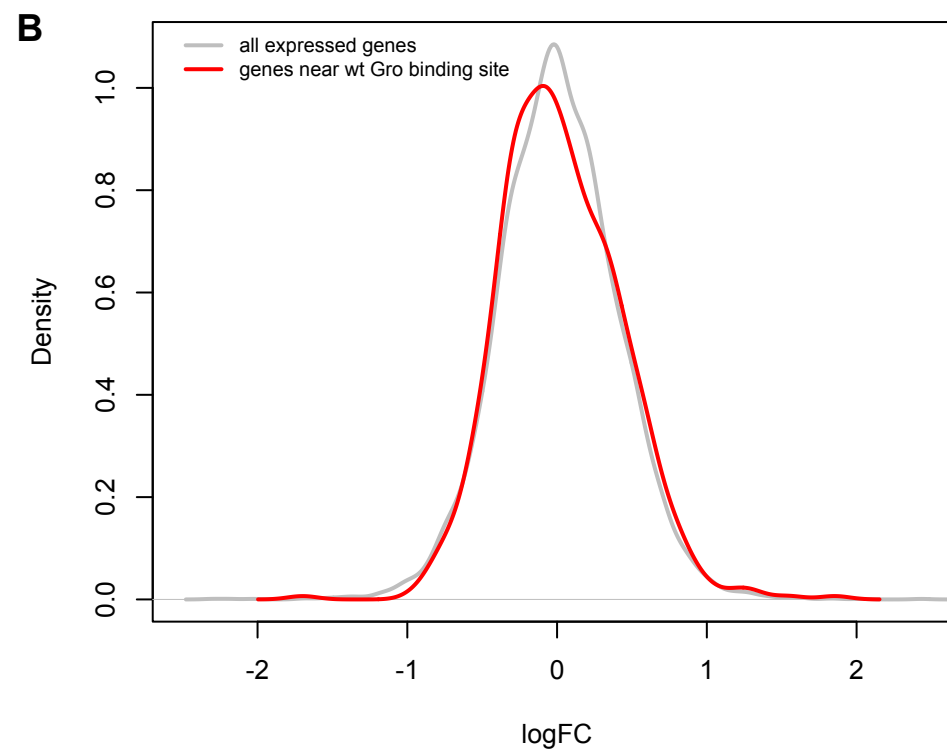

Supplement: Figure S6 — Gene expression profiles of untreated and gro RNAi treated S2 cells. A) Plot illustrating the log fold changes (logFC) for all expressed genes (grey) and with genes mapping nearest to a Gro binding site (red). B) Density plot illustrating the distribution of log fold changes (logFC) for all expressed genes and genes nearest to a Gro binding site. (PDF) [file pgen.1004595.s006.pdf]

**A**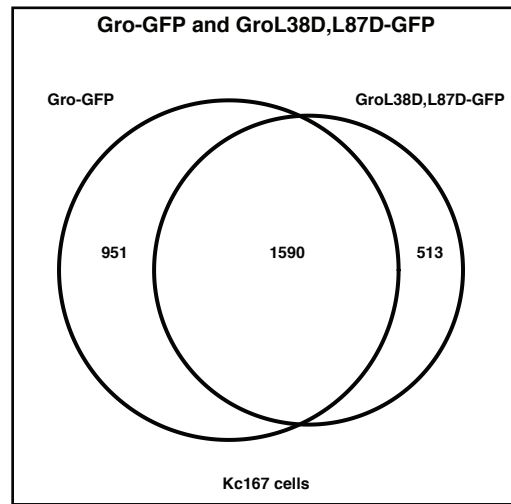**B**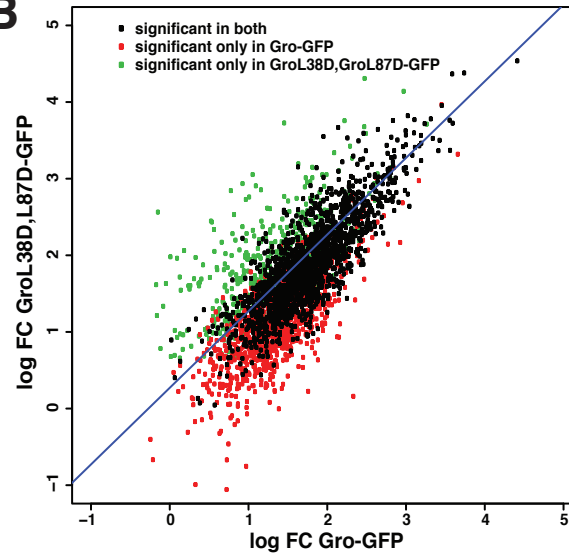**C**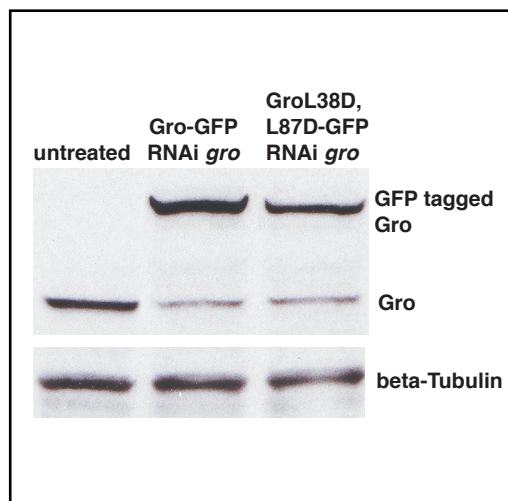

Supplement: Figure S7 — Characterization of Gro-GFP and GroL38D,L87D-GFP recruitment and expression in Kc167 cells. A) Venn diagram showing the overlap between Gro-GFP and GroL38D,L87D-GFP peaks in Kc167 cells. B) Plot showing the log fold change (FC) of Gro-GFP and GroL38D,L87D-GFP peaks (100 bp fragment nearest the summit of each peak) in Kc167 cells after normalization in edgeR [68]. C) Western blot analysis showing the expression of endogenous Gro and GFP-tagged Gro variants (detected by anti-GFP antibody) in untreated and treated Kc167 cells as indicated, with beta-Tubulin included as a loading control. (PDF) [file pgen.1004595.s007.pdf]

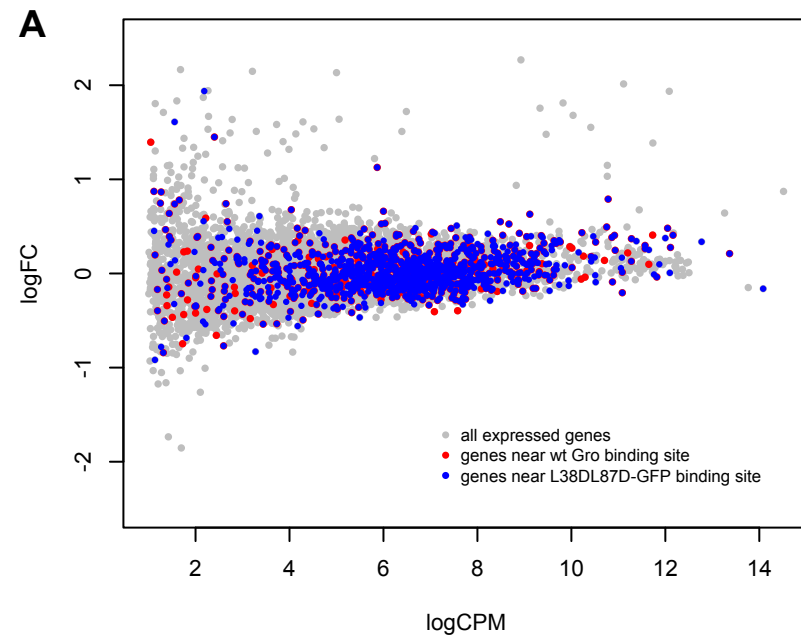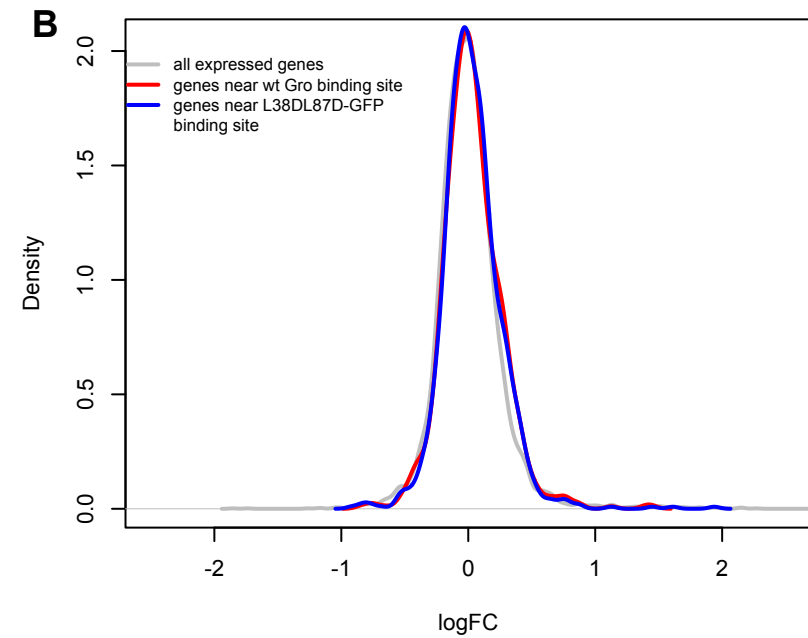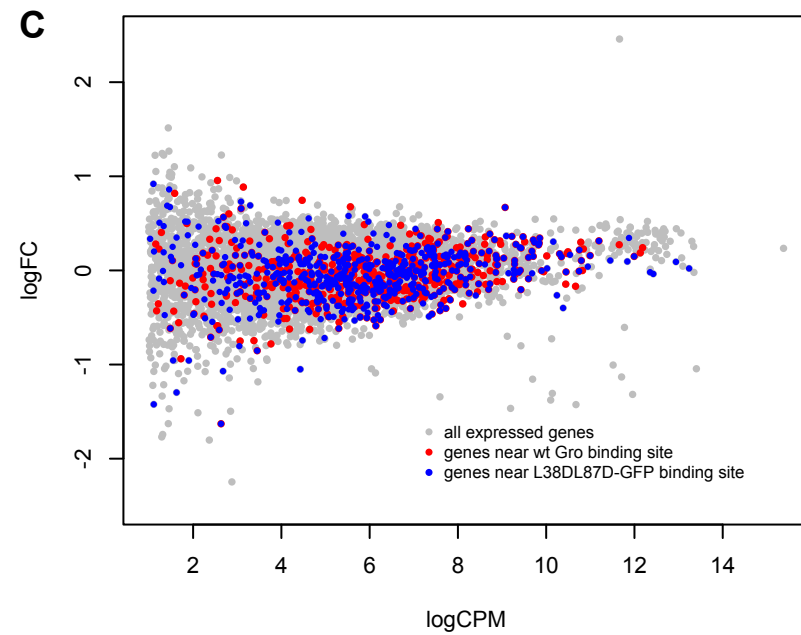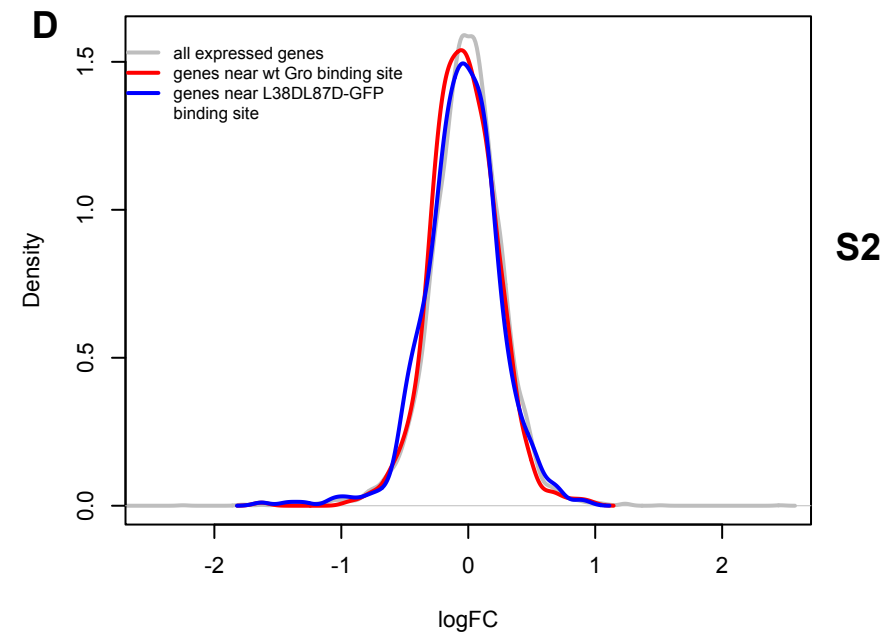

Supplement: Figure S8 — Comparison of gene expression in cells expressing Gro-GFP and L38D,L87D-GFP by RNA-seq analysis. A) Plot illustrating the log fold changes (logFC) for all expressed genes (grey), genes mapping nearest to a Gro binding site (red) and genes mapping nearest to peaks bound by L38D,L87D-GFP (blue) in Kc167 cells. B) Density plot illustrating the distribution of log fold changes (logFC) for all expressed genes (grey), genes nearest to a Gro binding site (red) and genes nearest L38D,L87D-GFP peaks (blue) in Kc167 cells. C) Plot illustrating the log fold changes (logFC) for all expressed genes (grey), genes mapping nearest to a Gro binding site (red) and genes mapping nearest to peaks bound by L38D,L87D-GFP (blue) in S2 cells. D) Density plot illustrating the distribution of log fold changes (logFC) for all expressed genes (grey), genes nearest to a Gro binding site (red) and genes nearest L38D,L87D-GFP peaks (blue) in S2 cells. (PDF) [file pgen.1004595.s008.pdf]

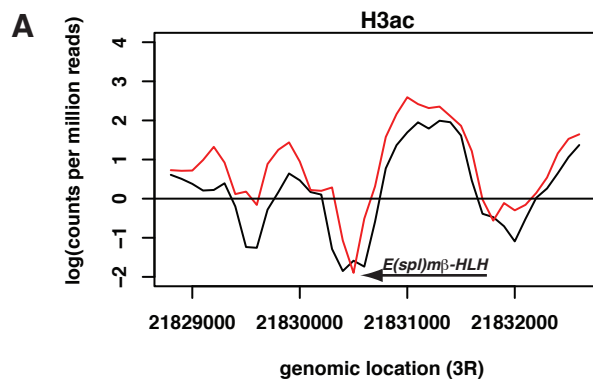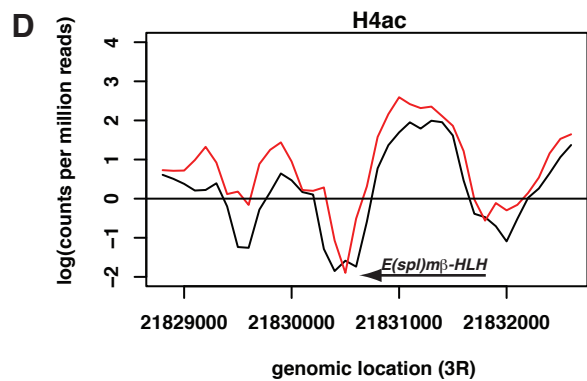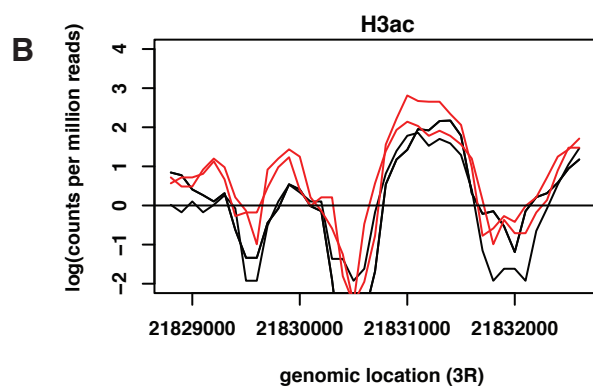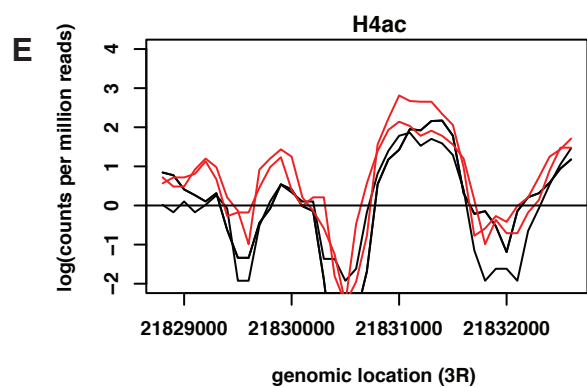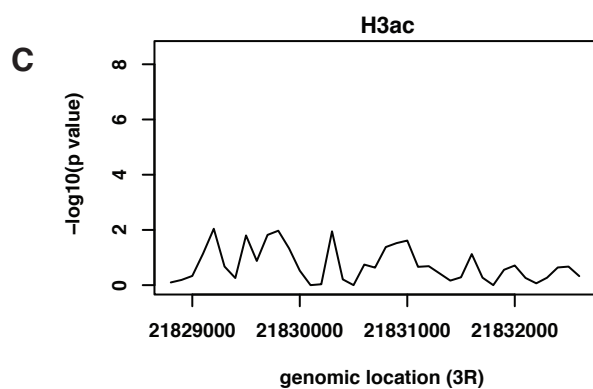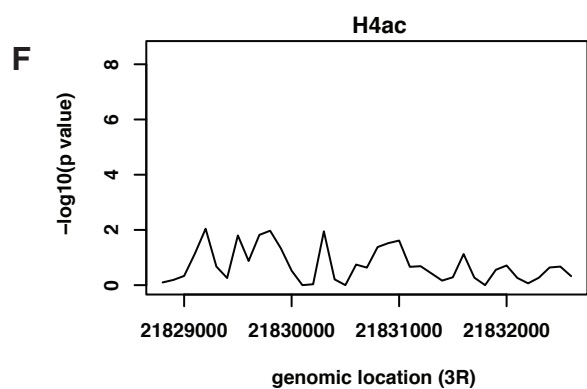

Supplement: Figure S9 — Profiles of Histone H3 and H4 acetylation at the E(spl)mβ-HLH locus in Kc167 cells. A) Plot of average level of H3 acetylation across the E(spl)mβ-HLH locus from untreated cells (black) and cells treated with gro RNAi (red). Profiles were taken from the average normalized counts of 100 bp fragments from an analysis in edgeR [68]. B) Plot of the individual replicate samples used to make the plots in A (after normalization). C) Regions that are significantly different between untreated and gro RNAi samples for histone H3 acetylation. Note: this shows −log10 (p value) peaks. D) Normalized plot of H4 acetylation across the E(spl)mβ-HLH locus from untreated cells (black) and cells treated with gro RNAi (red). E) Plot of the individual replicate samples used to make the plot in D (after normalization). F) Regions that are significantly different between untreated and gro RNAi samples for histone H4 acetylation. Note: this shows -log10 (p value) peaks. (PDF) [file pgen.1004595.s009.pdf]

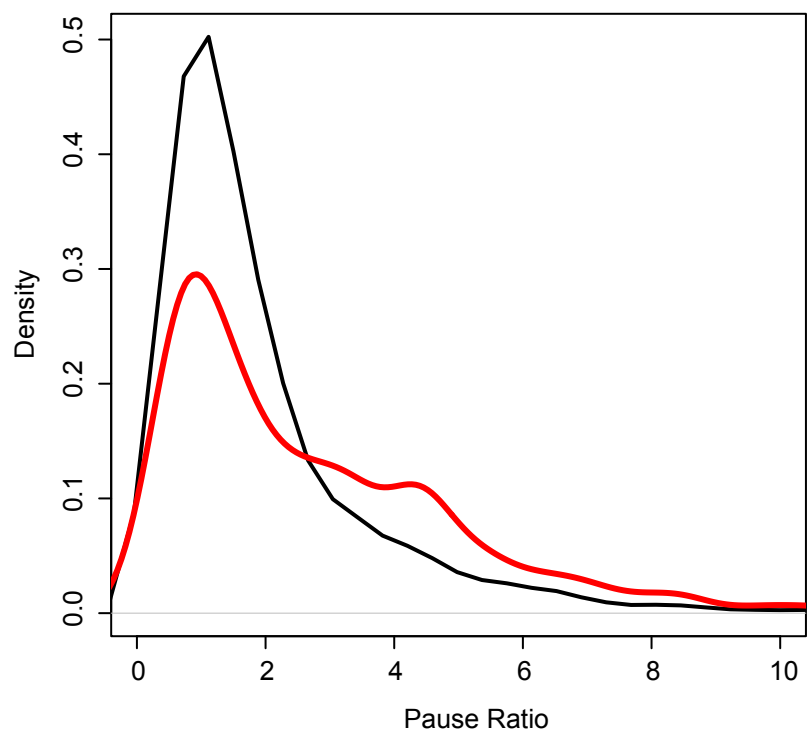

Supplement: Figure S10 — Gro is enriched at paused transcripts. Density plot showing the pause ratio of all transcripts (black) and transcripts associated with Gro binding (red). (PDF) [file pgen.1004595.s010.pdf]
