# Supplementary material for: The Groucho Co-repressor Is Primarily Recruited to Local Target Sites in Active Chromatin to Attenuate Transcription
Source: PLoS Genet. 2014 Aug 28;10(8):e1004595. doi: 10.1371/journal.pgen.1004595 (PMC4148212; doi:10.1371/journal.pgen.1004595)
Supplement: Protocol S1 — Additional details of the materials and methods for ChIP-seq experiments. (DOCX) [file pgen.1004595.s011.docx]

**PROTOCOL S1**

**Cell culture and transfection**

Kc167 and S2R+ cells (DGRC) were grown in Schneider's Insect Cell Medium (GIBCO) supplemented with 10% FBS (Sigma) and 1% penicillin/streptomycin (Thermoscientific) and kept at 25°C. Cells were transfected with dsRNA against the 3’UTR of *gro* and plasmids containing either Gro-GFP or GroL38D,L87D-GFP. Transfections were performed using Effectene according to manufacturer’s instructions (Qiagen). 300-600ng DNA and 1 μg dsRNA were transfected per 1-3 x 10^6^ cells. Successful transfection and knockdown were assessed by western blot.

**Western blot analysis**

Cells were lysed six days after transfection, denatured in 1xNuPage sample buffer (Life Sciences), and electrophoresed on a polyacrylamide gel. Following transfer to a nitrocellulose membrane (Amersham Hybond ECL - GE Healthcare), proteins were detected by immunoblotting and subsequently measured semiquantitatively with ECL-Plus (GE Healthcare) and Kodak BioMax MR Film. Blots were probed with mouse anti-Gro (1:20; DSHB) and ß-tubulin (1:2000; DSHB). The latter served as a loading control.

**Chromatin Immunoprecipitation (ChIP)**

For Gro and GFP ChIP experiments, cells were double crosslinked by incubation with Disuccininmidyl glutarate (DSG-Fisher Scientific; 2mM) for 20 minutes on a rocker followed by treatment with 1% formaldehyde for ten minutes. For all other ChIP experiments, cells were single crosslinked by incubation with 1% formaldehyde for ten minutes. The fixation reaction was quenched with 200mM Glycine after which cells were washed three times with 1 x Phosphate Buffered Saline (1 x PBS). This was followed by a ten-minute lysis on ice (Nuclear Lysis Buffer: 50mM Tris-HCL pH 8.1, 10mM EDTA, 1% SDS, 1 x Roche Complete Inhibitor) and the addition of 1.6 volumes of IP Dilution Buffer (20mM Tris-HCL pH 8.1, 150mM NaCl, 2mM EDTA, 1% TritonX-100, 0.01% SDS, 1 x Roche Complete Inhibitor).

Chromatin released from lysates was fragmented to a range of 200 to 1000bp using a Diagenode Bioruptor set for 20 pulses (30 seconds ON, 30 seconds OFF) for double crosslinked samples and 15 pulses (30 seconds ON, 30 seconds OFF) for single crosslinked samples. Fragmented chromatin was further diluted (five-fold) in IP dilution buffer and precleared for 3-5 hours with Protein G PLUS-Agarose beads and 3 μg of normal rabbit IgG (Insight Biotechnology and New England Biolabs). Following this precleared chromatin was either snap-frozen and stored at minus 80°C or immediately used for ChIP.

For each ChIP sample an aliquot of chromatin deriving from approximately 1 x 10^7^ cells was used. For immunoprecipitation the following antibodies and concentrations were used: anti-Gro antibody KW01 (a gift from Kevin White) at 1 in a 100; GFP rabbit (VWR International) at 1 in 500; anti-acetylated histone H3 (Millipore) at 1 in 50; anti-acetylated histone H4 (Millipore) at 1 in 75; anti-RNA polymerase II (phospho S2) (VWR International) at 1 in 20; anti-Rpb3 (a gift from Karen Adelman) at 1 in 50. To normalize ChIP experiments an aliquot was kept aside to act as an input control. A further sample was treated with Normal rabbit IgG (at 1 in 250) to act as a negative control. Antibodies were added along with Protein G PLUS-Agarose beads and samples were kept on a rocker at 4°C overnight.

After immunoprecipitation the beads were subjected to a wash with IP Dilution Buffer and two washes each with IP Wash Buffers 1 and 2 [IP Wash Buffer 1: 20mM Tris-HCL (pH 8.1), 50mM NaCl, 2mM EDTA, 1% TritonX-100, 0.1% SDS; IP Wash Buffer 2: 10mM Tris-HCl (pH 8.1), 250mM LiCl, 1mM EDTA, 1% NP-40, 1% deoxycholic acid)]. They were finally washed with TE Buffer before bound chromatin-protein complexes were eluted (IP Elution Buffer: 100mM NaHCO_3_, 1% SDS). Eluted complexes were treated with RNase A (Invitrogen) at 37°C for an hour to eliminate RNA, 180mM NaCl at 65°C for 4-6 hours to reverse crosslinks, and Proteinase K (Roche) at 45°C overnight to remove proteins. The aliquot kept aside as input was subjected to the same treatment. Purified DNA for ChIP and input was obtained by phenol-chloroform extraction and ethanol precipitation. The concentration of ChIP and Input DNA was checked on the Qubit fluorometer (Invitrogen).

Following ChIP 20-50 ng of each sample was used to create a ChIP library for sequencing on the MiSeq Illumina platform following manufacturers’ instructions [NEBnext ChIP-Seq Kit (E6240S) or NEBNext Ultra DNA Library Prep Kit (E7370S)]. Adaptor-ligated DNA fragments of 250-350 bp were selected via gel extraction and purification. Paired-end 25 bp reads were generated for all Gro and GFP ChIP and input samples and single-end 50 bp reads were generated for all other samples. For all samples two biological replicates were sequenced.

For all Gro and GFP samples at least 2.9 million uniquely aligned reads were generated per replicate and for all other samples at least 7 million reads were generated per replicate. This is above the minimum number recommended by modENCODE project guidelines for *Drosophila* "point" and "broad" source factors respectively [62].

For RNA-seq experiments total RNA was obtained from approximately 5 x 10^6^ cells per sample (Qiagen RNeasy mini kit). mRNA was then extracted using the Dynabeads® mRNA Purification Kit (Life Technologies). 50-250 ng of mRNA was used to generate an mRNA library following manufacturers’ instructions (NEBnext mRNA Library Prep Master Mix – E6110S). Adaptor-ligated cDNA fragments of 250-350 bp were selected and processed as for ChIP libraries. Samples were sequenced on the Illumina MiSeq following the manufacturer's protocol and paired-end 36 bp reads generated. For all samples two biological replicates were sequenced and at least 7 million reads generated per replicate.
